# Managing flood risks in the Mekong Delta: How to address emerging challenges under climate change and socioeconomic developments

**DOI:** 10.1007/s13280-017-1009-4

**Published:** 2018-02-24

**Authors:** Long Phi Hoang, Robbert Biesbroek, Van Pham Dang Tri, Matti Kummu, Michelle T. H. van Vliet, Rik Leemans, Pavel Kabat, Fulco Ludwig

**Affiliations:** 1 Water Systems and Global Change Group, Wageningen University, Droevendaalsesteeg 3, 6700 AA Wageningen, The Netherlands; 2 Public Administration and Policy Group, Wageningen University, Hollandseweg 1, 6706 KN Wageningen, The Netherlands; 3 College of Environment and Natural Resources, Can Tho University, 3/2 Street, Ninh Kieu District, Can Tho, Vietnam; 4 Water & Development Research Group, Aalto University, P.O.Box 15200, 00076 Aalto, Finland; 5 Environmental Systems Analysis Group, Wageningen University, Droevendaalsesteeg 3, 6700 AA Wageningen, The Netherlands; 6 International Institute for Applied Systems Analysis, Schlossplatz 1, 2361 Laxenburg, Austria

**Keywords:** Challenges, Climate change, Flood-risk management, Mekong Delta, Socioeconomic developments, Solutions

## Abstract

Climate change and accelerating socioeconomic developments increasingly challenge flood-risk management in the Vietnamese Mekong River Delta—a typical large, economically dynamic and highly vulnerable delta. This study identifies and addresses the emerging challenges for flood-risk management. Furthermore, we identify and analyse response solutions, focusing on meaningful configurations of the individual solutions and how they can be tailored to specific challenges using expert surveys, content analysis techniques and statistical inferences. Our findings show that the challenges for flood-risk management are diverse, but critical challenges predominantly arise from the current governance and institutional settings. The top-three challenges include weak collaboration, conflicting management objectives and low responsiveness to new issues. We identified 114 reported solutions and developed six flood management strategies that are tailored to specific challenges. We conclude that the current technology-centric flood management approach is insufficient given the rapid socioecological changes. This approach therefore should be adapted towards a more balanced management configuration where technical and infrastructural measures are combined with institutional and governance resolutions. Insights from this study contribute to the emerging repertoire of contemporary flood management solutions, especially through their configurations and tailoring to specific challenges.

**Electronic supplementary material:**

The online version of this article (10.1007/s13280-017-1009-4) contains supplementary material, which is available to authorized users.

## Introduction

**Fig. 1:**
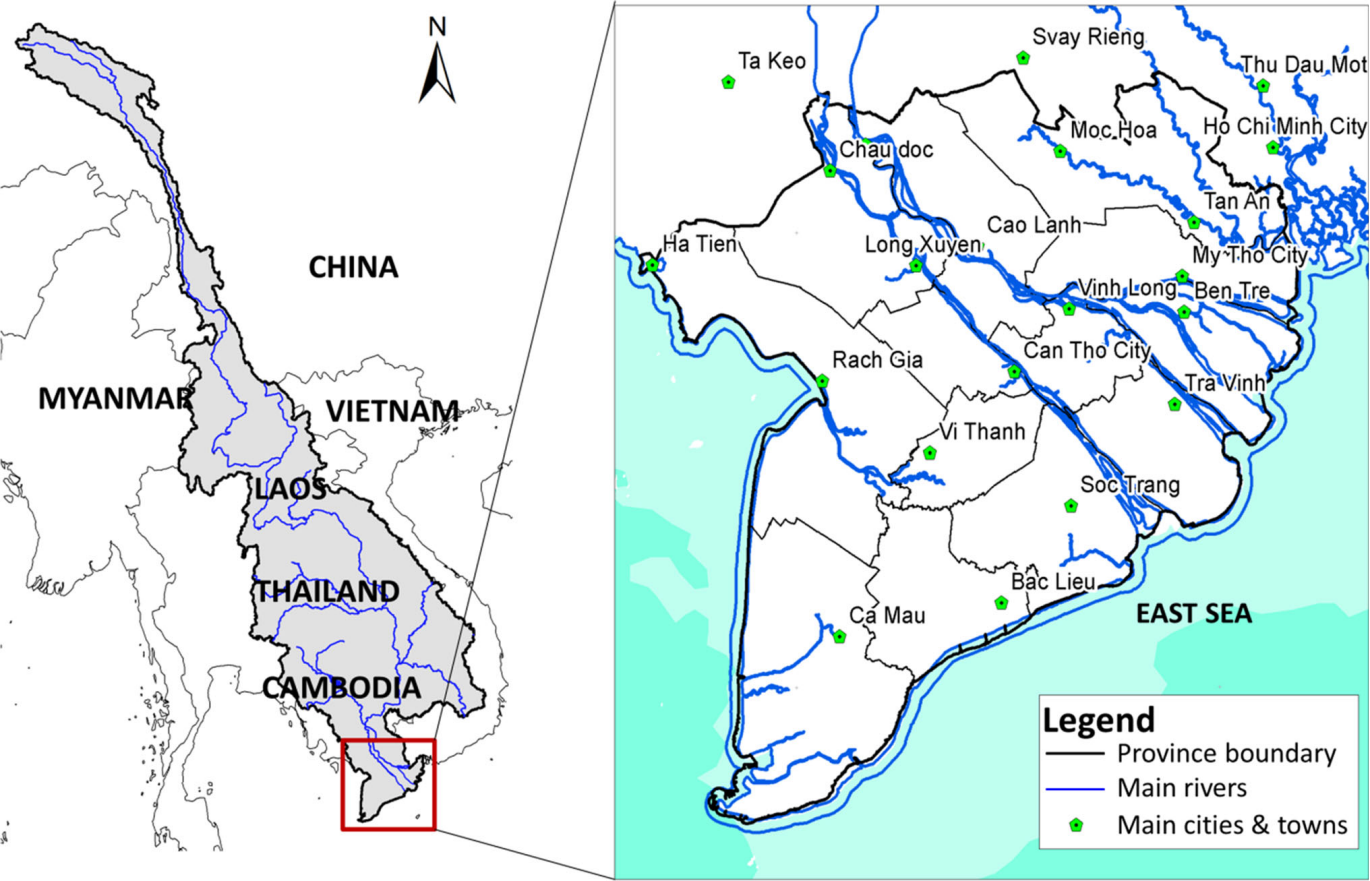
Overview maps of the Mekong River Basin (left) and the Mekong Delta (right)

Annual floods in the Vietnamese Mekong River Delta not only bring great benefits for local inhabitants and the regional economy but also constitute a major safety risk (Hoa et al. 2008; MDP 2013). Located in the downstream reach of the Mekong River (Fig. 1), the Mekong River Delta (hereafter, the Mekong Delta) receives about 475 km^3^ of upstream inflow annually (MRC 2005). About 70–80% of this flow volume comes during the wet season (July–December), causing widespread flooding across the floodplains. Floodwater, especially the overland water flow, generates multiple benefits for natural ecosystems, fisheries and agriculture (Arias et al. 2013; Chapman et al. 2016). These benefits include providing migration routes and breeding sites for fish species, distributing nutrient-rich sediment for agriculture, recharging ground water aquifers and controlling sea-water intrusion. Despite these abundant benefits, extreme floods also cause losses of human lives and severe damages to crops and infrastructures (Västilä et al. 2010; Van et al. 2012). For example, the historic flood in 2000, a 50-year flood with estimated economic losses of over US$ 200 million, illustrates the delta’s high vulnerability to extreme floods (Cosslett and Cosslett 2014). Given the valuable benefits and severe flood damages, flood management in the Mekong Delta requires effectively controlling excessive floodwater without compromising the flood benefits and other development objectives (Käkönen 2008; Pham 2011).

Flood management in the Mekong Delta, however, is facing critical challenges caused by climate change and accelerating socioeconomic developments (MDP 2013). Challenges are defined here as factors or processes that can hinder successful planning and implementation of flood management activities. Flood hazards are projected to increase substantially under future climate change due to higher upstream inflow and downstream sea-level rise (Wassmann et al. 2004; Nguyen et al. 2014). These increasing flood hazards are expected to exceed the delta’s current coping capacity and thus constitute a major threat for safety and sustainable development (Thanh et al. 2004; Wassmann et al. 2004). Furthermore, prevalent uncertainties in the future flood hazards also hamper long-term planning and investments for flood management (MDP 2013; Trung and Thanh 2013). Accelerating socioeconomic developments including economic and population growths, land-use change and infrastructural developments (e.g. building dikes and hydropower dams) also introduce new management challenges. Population in the Mekong basin increased from 63 million to 72 million during the 1995–2005 period, and a further increase of 60% is projected by 2050 (Pech and Sunada 2008). The agriculture sector also experienced similar trends, with irrigated rice area increasing more than three times during 1975–1994 in the An Giang Province—one of the development hot spots in the Mekong Delta (Käkönen 2008). All these developments will likely exert extra pressures on the Mekong Delta’s water system including flood risks.

Since the launch of the “*Doi Moi”* policy^1^ (Pham 2011) during the early 1990s, the delta’s economic structure has developed from a rice-based economy towards a more diversified system with growing contributions from fishery, aquaculture, horticulture, services, trade and industry (Huynh et al. 2008; Thai et al. 2008). This diversified economy requires pursuing multiple, sometimes competing, flood management objectives (Käkönen 2008; Renaud and Küenzer 2012). Reflecting on these objectives, Käkönen (2008) and Pham (2011) questioned the suitability of the current technology-centric flood management approach to spontaneously secure flood safety and sustain flood benefits. This and other challenges experienced in flood management were also reported in the recent literature, including technical difficulties (Hoa et al. 2008; MDP 2013), limited resources and capacity (Bastakoti et al. 2014; Hoa et al. 2014a), and governance and institutional constraints (Waibel et al. 2012; MDP 2013). Without timely solutions, the challenges can hamper flood management efforts, thereby creating serious consequences for the people and the economy of the Mekong Delta (MDP 2013).

**Fig. 2:**
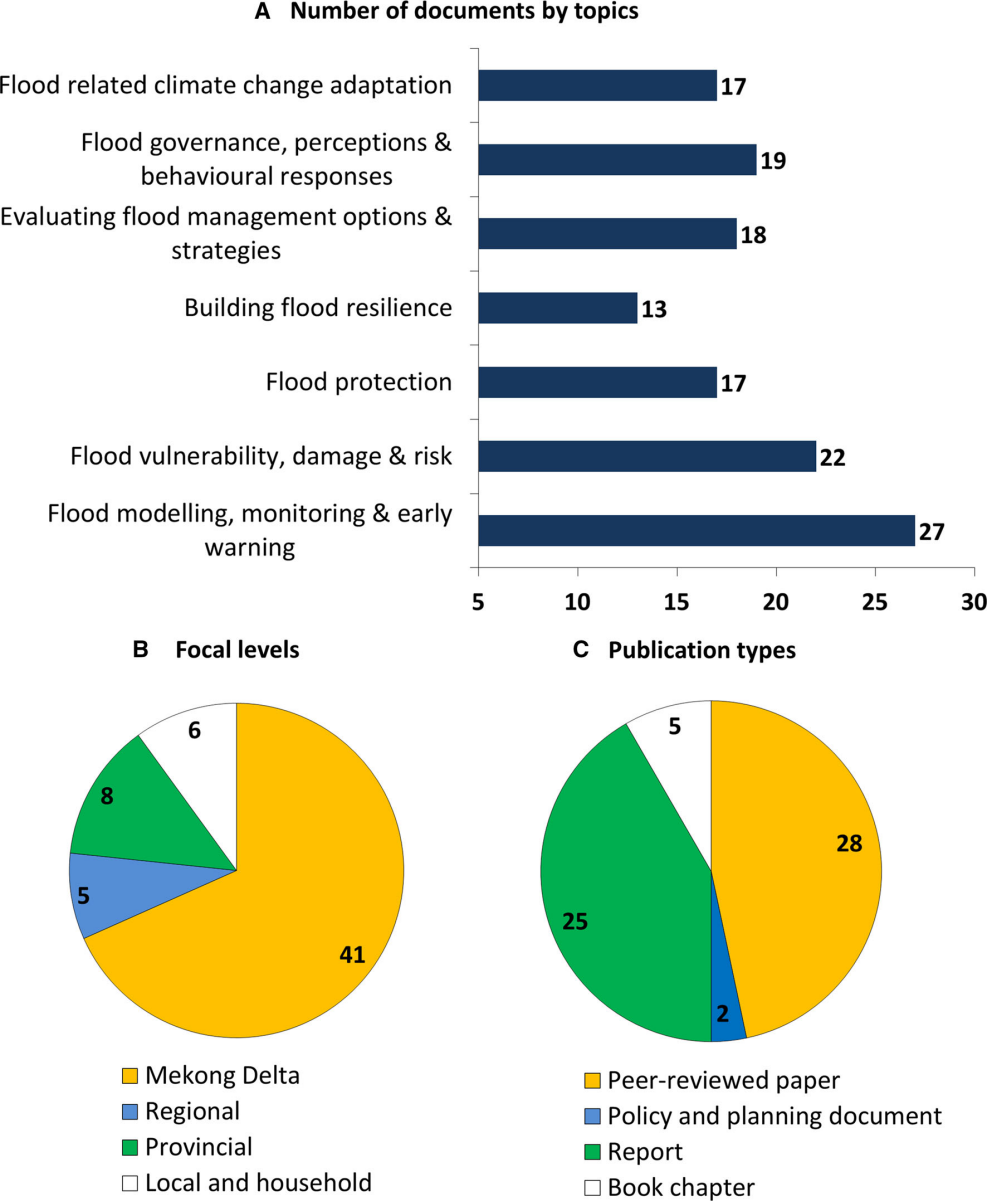
Compositional profile of the reviewed literature

Despite numerous studies on flood risks and management in the Mekong Delta (see an overview of recent studies in Supplementary Material S1), little attention is paid to the recently emerging challenges for flood management under climate change and accelerating socioeconomic developments. In many cases, emphasis is still placed on finding the ‘right’ technical solutions, following the conventional flood management approach (e.g. Lebel and Sinh 2009; Marchand et al. 2014). The number of flood management studies explicitly including climate change and socioeconomic developments, on the other hand, remains limited (see also "Systematic literature review" section on literature review and Fig. 2A). As a result, the questions of which challenges are more critical in the changing flood management context and how to effectively overcome them using a mix of different types of solutions (i.e. strategies) remain largely unaddressed. In addition, little is known about how existing challenges manifest and to what extent new challenges arise due to climate change and socioeconomic developments. These important knowledge gaps need to be addressed to effectively inform and support flood management in the Mekong Delta to deal with climate change and rapid socioeconomic developments.

This study therefore aims to (i) systematically identify the key challenges for flood management in the context of climate change and accelerating socioeconomic developments, and (ii) identify intervention solutions and develop strategies to adequately address these challenges for the Mekong Delta. We collected data using a systematic literature review and implemented expert surveys ("Systematic literature review" and "Expert survey" sections). Using statistical inferences and qualitative data analysis techniques ("Data analysis" section), we identify and analyse a diverse set of flood management challenges ("Current flood management approach and key challenges" section). We present 114 identified solutions and six thematic strategies to address the key flood management challenges ("Solutions and strategies to address flood management challenges" section). In "Tailoring strategies and solutions to flood management challenges" section, we describe how the strategies and solutions are tailored to the challenges as guidance for implementation. "Discussion" section discusses the results, their implications for flood management, and "Conclusion" section concludes.

## Materials and Methods

### Systematic literature review

We used systematic review methods (Biesbroek et al. 2013; Ford et al. 2015) to collect and analyse all relevant peer reviewed literature using the ISI Web of Science Database. The database search used “Mekong”, “Delta” and “flood” as keywords and this query returned 141 entries, from which we selected 94 documents and excluded 47 irrelevant documents (based on their titles). The search keywords were kept generic and broad, with the intention to capture all relevant sub-topics such as climate change impacts, risks or vulnerability in relation to floods. We were also interested in other relevant documents that are not available in this database including grey literature such as policy and planning documents and those published in Vietnamese. We cross-checked our findings using expert deliberation methods (Petticrew and Roberts 2008) to retrieved 19 additional documents. In total, the literature search yielded 113 documents, which were subjected to a detailed screening procedure based on relevance and content. This further eliminated 53 documents, because they either did not cover our study area, or did not relate to the flood management topic. The complete procedure resulted in 60 relevant documents, which were included into the detailed literature review and analyses.

To structure the analysis, we extracted relevant information from the collected documents into a data extraction table: (1) Generic information (authors, publication year, publication type, topic and geographical coverage); (2) Flood management challenges reported in the study (further classified into Group 1—technical challenges, Group 2—institutional and governance challenges, and Group 3—resources and capacity challenges). See Supplementary Material S1 for the review protocol and data extraction table.

#### Literature profile

The total 60 documents (Supplementary Material S1) consist of 28 peer-reviewed scientific articles, 5 book chapters, 25 reports and 2 planning and policy documents. The focal topics, spatial levels and publication types of the reviewed literature are summarized in Fig. 2. All the reviewed documents date between 2000 and 2017. Topic-wise, the literature exhibits relatively equal coverages of different flood management aspects. Flood modelling, monitoring and early warning are most frequently reported (*n* = 27) while building flood resilience topic shows the lowest coverage (*n* = 13). Regarding spatial levels, a majority (*n* = 41) of the included documents focuses on the delta-wide level. Flood management at the sub-delta levels (i.e. regional, provincial, local and individual households), however, receives less attention, shown by markedly fewer documents.

### Expert survey

In the second step, we used the main findings from the literature review to design a survey to collect insights from relevant experts about two key questions: (1) What do they consider to be the key challenges for flood management in the Mekong Delta?; and (2) What do they consider as the suitable solutions to overcome specific challenges? The survey combines multiple-choice and open-ended questions to collect information about flood management challenges, potential solutions and the experts’ professional backgrounds (see Supplementary Material S2-a for the survey).

The survey is self-administrated and is implemented via an online survey platform (LimeSurvey 2015). Survey respondents were identified from the authors’ research networks, contact information found in the relevant literature and secondary referrals (i.e. respondents introduce new experts who they think are suitable for the survey). The online-survey strategy helps effectively targeting many respondents within reasonable survey administration time. Also, this strategy is especially useful given that our targeting respondents are spread out in different locations (Kumar 2005). In total, the survey invitation was sent via email to 132 experts, followed by two reminders sent after 2 and 4 weeks, respectively.

#### Expert sample

In total, 71 out of 132 invited experts completed the survey. They consist of 14 government officers, 13 NGO officers or consultants, 22 natural scientists, 13 social scientists, 7 engineers and 2 experts with other occupations. The respondents work at different spatial levels, ranging from local and provincial (*n* = 15), delta-wide (*n* = 27), to national (*n* = 11) and international (*n* = 18). They work on various flood-relating topics, including flood research (*n* = 14), water management and planning (*n* = 18), land-use management and planning (*n* = 5), flood protection (*n* = 2), building flood resilience (*n* = 12), and climate change impact and adaptation (*n* = 12). About one-third of the experts (i.e. 21 out of 71) listed flood as the central focus of their professional practices. Overall, the expert sample shows relatively good representations of both spatial levels and flood management aspects.

### Data analysis

We used both quantitative and qualitative data analysis techniques to gain insights about various aspects, including the literature profile, expert sample, flood management challenges, solutions and strategies. We first analysed the compositional characteristics of the literature and the expert sample by calculating standard descriptive statistics (i.e. sums, means and percentages). We calculated the expert sample’s composition by professional occupations, focal flood management aspects, and working levels.

We ranked the challenges by their levels of importance assigned by our expert panel. We converted the 5-level Likert scale to numeric values (1—Very unimportant to 5—Very important) following Kumar (2005) and further calculated the aggregate rankings for different expert groups. We also checked the linkages between the individual challenges by calculating correlation coefficients between the challenges’ rankings. In addition, we used multivariate regression to analyse how the respondents’ backgrounds (e.g. occupations, working levels and working focuses) influence their judgements about the challenges’ importance (Hoa et al. 2014b). Equations for calculating the above described statistics are available in Supplementary Material S2-b.

We used content analysis of the respondents’ open-ended responses to identify solutions and develop strategies to address flood management challenges (Kumar 2005; Biesbroek et al. 2011). The solutions were identified from the recommendations through open-coding technique, using Atlas-ti-v7 software. During open-coding, the respondents’ recommendations were summarized and systematically assigned to a set of codes (i.e. the codebook) where each code represents a flood management solution. The codebook was cross-validated following Kumar (2005). The coding procedure was quality-checked by comparing the solution sets derived from two independent coding exercises conducted by two of the authors, and all documents were recorded using the final codebook to ensure validity of the findings. After this, we analysed individual solutions based on their objectives and developed thematic flood management strategies (i.e. combinations of different individual solutions). Lastly, we calculated the recommendation rates (i.e. how many times a strategy is recommended for a particular challenge) to gain insights about how the strategies are tailored to different challenges according to the experts.

## Results

### Current flood management approach and key challenges

#### Current flood management approach

The systematic literature review revealed a wide variety of flood management solutions being implemented in the Vietnamese Mekong River Delta. While the currently practiced solutions show multiple aspects of flood-risk management, the predominant approach is flood prevention using infrastructural measures (see also MDP 2013; Marchand et al. 2014). In particular, the floodwater levels and flood extents are controlled by using a complex system of drainage, floodwater discharge canals, sluice gates and protection dikes (Pham 2011; Marchand et al. 2014). High dikes are used to protect residential areas and the main agricultural zones, while the secondary dikes protect crops against moderate floodwater levels at the beginning of the flood season. In addition to the main flood prevention solutions, current literature also reports different complementary solutions focusing on technical and regulatory aspects. The commonly implemented technical solutions include flood monitoring; early warning; flood emergency response plans; and communication and awareness raising (Trung et al. 2013; Hoa et al. 2014a). Several regulatory solutions are also practiced, including relocation from flood-prone zones, adaptation to flood and developing flood management legislations (Pham 2011). While the above described flood management solutions are being developed simultaneously, they are often implemented separately and show few interlinkages. As a result, the current flood management approach exhibits important fragmentations, where coordination and joint effort for implementation between regions and actors remain very limited. The following sections further demonstrate important consequences caused by such a fragmented flood management approach and subsequently identify suitable solutions.

#### Flood management challenges

We identified 19 flood management challenges (C1–C19) from the literature and further verified them with our expert panel (Table 1). Overall, the identified challenges are diverse and relate to different flood management aspects. They were grouped (G1–G3) into G1—Technical challenges (C1–C7); G2—Governance and institutional challenges (C8–C13); and G3—Resources and capacity challenges (C14–C19). Technical challenges (Group G1) are reported more often in the literature compared to the other groups, shown by a higher number of challenges and more reporting documents. The more frequently reported challenges in this group include “*C1*—*Lack of knowledge and understandings about the flood mechanisms in the floodplain* ”; “*C2*—*Existing flood protection measures create unwanted impacts*”; “*C4*—*Research results are not taken up in flood management*” and “*C7*—*Uncertainties in future climate change, sea*-*level rise and socioeconomic development hinder development of flood management plans*”. Various flood management challenges relating to the governance and institutional settings (Group G2) were also reported, resulting in the following main challenges: “*C9*—*Limited coordination and collaboration in flood management across provinces and districts*” and “*C10*—*Conflicting interests between different management departments and regions*”. Group G3 consists of challenges relating to resources and capacity for flood management. The commonly reported challenges in this group are “*C14*—*Flood management lacks financial resource*” and “*C18*—*Lack of data and equipment for flood risk management*”. We further found that flood management challenges in the Mekong Delta tend to relate to each other, shown by relatively high correlation coefficients between individual challenges (see Supplementary Material S2-c—Correlation coefficients between the challenge’s rankings). The strongest correlating challenges include C5, C9, C11, C15 and C19. These strong correlations suggest that the challenges exhibit intricate interlinkages and that they are often experienced together rather than individually in practice.

**Table 1:** Flood management challenges identified from systematic literature review. More details about the challenges and reporting literature is available in Supplementary Material S1. Numbers correspond to the reviewed documents listed in Supplementary Material S1

Challenges	Reporting literature
G1 Technical challenges	
C1 Lack of knowledge and understandings about the flood mechanisms in the floodplain	1–4, 6, 8, 11, 13–15, 18– 24, 27, 28, 30, 33, 35, 40, 42, 45–48, 51, 53–57
C2 Existing flood protection measures create unwanted impacts	6, 7, 9, 11, 16, 21, 24, 26, 28, 31, 32, 37, 40, 42, 46, 48, 52, 55, 58
C3 Flood forecasting and early warning systems are not effective and reliable	1, 17, 18, 25, 43, 51, 52
C4 Research results are not taken up in flood management	1, 17, 18, 22, 25, 32, 44, 58
C5 Local, indigenous knowledge is underused in flood management	12, 17, 18, 32, 39, 43
C6 Suitable strategies and measures for flood management are not available	5, 8, 17, 18, 25, 28, 31, 49, 51, 53, 58, 60
C7 Uncertainties in future climate change, sea-level rise and socioeconomic development hinder development of flood management plans	2, 4, 11, 18, 22, 23, 25, 30, 33, 35, 40, 51, 53, 54
G2 Governance and institutional challenges	
C8 Some factors causing flood are outside management boundary, i.e. in other country, province or district	3, 15, 28, 42, 53, 58, 59
C9 Limited coordination and collaboration in flood management across provinces and districts	1, 5, 24, 34, 38, 41–43, 45, 52, 53, 57–59
C10 Conflicting interests between different management departments and regions	6, 7, 12, 15, 25, 26, 28, 34, 35, 42, 43
C11 Flood and water management plans at different levels are inconsistent, causing difficulties in implementation	8, 33, 35, 42, 43
C12 Top-down, centralized approach to flood management	31–35, 41–43
C13 Flood management system is not responsive to new issues and challenges	18, 25, 42, 45
G3 Resource and capacity challenges	
C14 Flood management lacks financial resource	1, 5, 17, 18, 20, 25, 32, 42, 45, 46, 49, 50, 53, 59
C15 Finance for flood management does not reach relevant regions and stakeholders	1, 5, 20, 41, 46, 50
C16 Flood management staffs lack important capacities	18, 25, 33, 34, 42, 58
C17 Insufficient number of staffs for flood management	34, 42
C18 Lack of data and equipment for flood-risk management	1, 10, 11, 15, 19, 20, 22, 24, 29, 30, 36, 43, 45, 46, 51, 57
C19 Lack of legislative and institutional capacities for flood management	1, 6, 24, 34, 41, 44, 58, 59

**Fig. 3:**
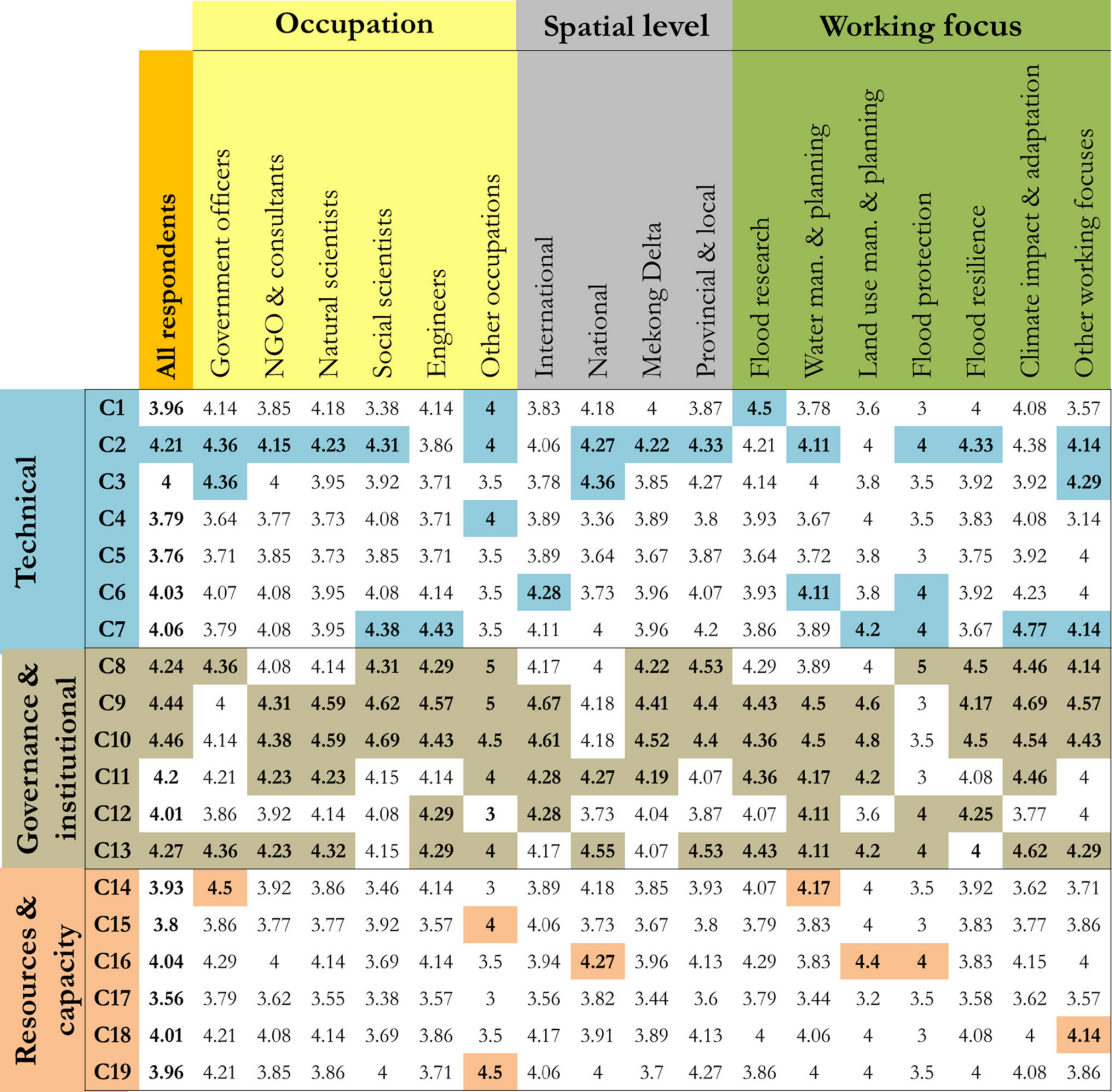
Ranking importance of flood management challenges (aggregated and per groups). Higher scores indicate more important challenges. Highlighted values indicate top-5 most important challenges according to each expert group (i.e. 5 highest values per column), whereas their colours correspond to three groups of challenges. C1–C19 refers to the challenges listed in Table 1

Results from the expert survey further indicate that many flood management challenges are considered to be very important (12 out of 19), and they tend to arise from the current governance and institutional settings in the Mekong Delta. Furthermore, 89% of the experts indicated that flood management has become more challenging comparing to three decades ago and they attribute the reasons to population growth (77%), dikes construction (70%), land-use change (68%), hydropower dam construction (68%), climate change (62%) and sea level rise (54%). In addition, experts’ evaluation clearly differentiated the challenges by their importance levels (Fig. 3). The top-five challenges according to all experts were: *C2*—*Existing flood protection measures create unwanted impacts*; *C8*—*Some factors causing flood are outside management boundary, i.e. in other country, province or district;* C9—*Limited coordination and collaboration in flood management across provinces and districts*; *C10*—*Conflicting interests between different management departments and regions*; and *C13*—*Flood management system is not responsive to new issues and challenges*. Notably, four out of the top-five challenges belong to group G2—governance and institutional challenges, making this group the most predominant one compared to the other groups. These challenges were consistently reported by experts from all occupations, working levels and working focuses, suggesting that they are commonly experienced across multiple spatial levels and at different aspects of flood management.

Some specific challenges (e.g. C2, C6 and C11) are found to manifest differently at multiple spatial levels, shown by their different important rankings across local, provincial, Mekong Delta, national and international levels. Ranking values for C2 and C11 (see Fig. 3) also show larger standard deviations between different spatial levels compared to the rest of the challenges. For example, the unwanted impacts of the current flood protection dikes (C2) were seen more important at the provincial and local levels. The dikes’ impacts, however, appeared less critical at the higher spatial levels, i.e. the Mekong Delta, national and international levels. Similarly, while challenge C11 (i.e. inconsistencies in planning) was considered important at the national and international levels, this challenges was regarded as less important at the provincial and local levels.

We also found that the rankings of several challenges (e.g. C2, C12, C13, C14 and C17) were dependent upon the expert’s occupation. For instance, the expert group of engineers did not consider the negative dike impacts (C2) as important, while all other groups regarded this challenge as a critical issue in the Mekong Delta. Differentiated rankings across the expert groups were also observed for C6 (lack of strategies and measures for flood management). Several respondent groups (i.e. engineers, internationally active experts and those working on water management and planning) regarded this challenge as highly important, whereas some other groups (i.e. those working at the national and Mekong Delta levels and natural scientists) did not see this as a critical issue. All in all, strong linkages between flood management challenges and their dependencies on local contexts (i.e. spatial levels and expert backgrounds) emphasize important implications for developing and implementing response solutions. These include the needs to integrate multiple solutions to address linked challenges, and to tailor the solutions to specific challenges taking into account local contexts.

### Solutions and strategies to address flood management challenges

We identified a relatively large set of flood management solutions from expert surveys and further analysed how these individual solutions can be configured into key thematic strategies for implementation. Overall, the identified solutions exhibit a remarkable degree of diversity in terms of their quantity (114 in total) and objectives. These solutions address different aspects of flood-risk management, ranging from infrastructural and technical interventions to mobilizing and developing capacities and resources. A complete inventory of solutions is presented in Supplementary Material S3-a. Despite this remarkable diversity, the identified flood management solutions also exhibit several generic patterns. First, the solutions show differentiated levels of priority for implementation, where certain solutions are recommended more often by the experts. The most frequently recommended solutions include “*Promote exchange and learning*”, “*Implement integrated flood impacts assessment*”, “*Improve collaboration between stakeholders*”, “*Improve communication*”, and “*Build capacity for flood management staffs*”. Second, while these top-prioritized solutions show a strong focus on management and capacity aspects, many infrastructural measures are also considered important for the Mekong Delta (see Table 2). Main infrastructural measures include “*Optimize the existing flood control infrastructures*”, “*Develop new technical measures for flood management*” and “*Address the unwanted impacts of existing flood management infrastructures*”. A relatively good mix of hard and soft solutions as shown in Table 2 emphasizes the importance of combining multiple solutions to address the increasing flood risks in the Mekong Delta.

**Table 2:** Main solutions to address the Top-five flood management challenges

Top challenges	Important rank	Ranking score	Solutions
C10 Conflicting interests between different management departments and regions	1st	4.46	Promote integrated management Promote multi-objective flood management Implement integrated flood impact assessment Improve data sharing Improve collaboration between actors
C9 Limited coordination and collaboration in flood management across provinces and districts	2nd	4.44	Develop coordinating board Improve collaboration between actors Promote exchange and learning Promote multi-level management Improve data sharing
C13 Flood management system is not responsive to new issues and challenges	3rd	4.27	Shift thinking and management paradigm Set priorities in management Improve communication Build capacity for flood management staffs Improve knowledge uptake
C8 Some factors causing flood are outside management boundary, i.e. in other country, province or district	4th	4.24	Improve collaboration between regions Improve collaboration between actors Improve communication Promote exchange and learning Implement integrated flood impact assessment
C2 Existing flood protection measures create unwanted impacts	5th	4.21	Revise existing measures Develop new technical measures Address unwanted impacts of existing measures Optimize existing control infrastructures Promote integrated planning

Motivated by the strong interlinkages between flood management challenges and the need for integrating response solutions, we further configured individual solutions into thematic strategies for implementation. Below the strategies are described together with their main solutions. The list of strategies and their associated solutions is provided in Supplementary Material S3-b (Flood management strategies and associated solutions).

#### Strategy S1: Create an enabling environment for flood management

A more enabling environment for flood management in the Mekong Delta entails three clusters of solutions. Firstly, the experts recommend a more participatory and inclusive flood management environment, where stakeholders can affectively participate in the process of planning and implementing management solutions. Representative solutions within this cluster include promoting participatory approaches and supporting stakeholder’s negotiation. The second cluster of solutions targets limited coordination in flood management. Here, improvements are needed for both cross-regional and between-stakeholders coordination. In response to the currently limited management coordination, many experts suggest establishing a coordinating board at the delta level. Lastly, resolving the current management bottlenecks constitutes the third solution cluster, with specific solutions include resolving conflicts; developing agreements and common understanding between stakeholders; and improving transparency in flood management.

#### Strategy S2: Strengthen and diversify the flood management portfolio

Overall, strategy S2 aims at developing a better flood management portfolio. Such portfolio is configured of multiple solutions which together ensure that flood management practices are (1) better integrated; (2) better tailored to the local contexts; and (3) more diverse. Commonly suggested solutions to pursue integrated flood management are promoting integrated flood management approaches; adapting multi-objective flood management; and combining multiple measures in planning and implementation. Tailoring flood management measures to the local context, on the other hand, can be achieved by localizing management processes, applying local knowledge and considering local conditions and resources availability when implementing the measures. Lastly, the experts suggest diversifying the current management portfolio with specific solutions including exploring flood benefits; using complementary measures to resolve unwanted impacts of the flood protection dikes; and developing non-regret and adaptive measures.

#### Strategy S3: Foster cross-boundary interactions

Strategy S3 is characterized by two main themes, namely collaboration; and exchange and learning. Experts strongly emphasize improving collaborations both across regions and between different stakeholders. Regarding the spatial aspect, inter-provincial collaboration through joint projects and data sharing is a frequently recommended solution. In addition, collaboration with upstream countries in the Mekong river basin is also often suggested, with specific solutions including participating in international forums; and improving the Mekong River Commission’s role in coordinating international dialogues and negotiations. The second aspect of cross-boundary interactions focuses on “Promoting exchanges and learning”, where specific solutions include organizing workshops, benefiting from international expertise and sharing experiences with similar river deltas. Overall, improved exchange and learning are recommended both within the Mekong Delta and at the international level.

#### Strategy S4: Improve capacity and resources

Improvements in capacity and resources for flood management are mostly recommended by improving financial and human resources. Besides a higher share of state budget for flood management, experts consider it to be necessary to diversify the financial resources through several specific solutions including combining loan and grant in project funding; generating funding through international collaboration; and attracting investment from the private sector. Regarding human resources, specialized training and education is strongly emphasized as a main solution to improve staff’s expertise and skills. In addition, improving recruitment effectiveness and better employment conditions are also regarded as suitable solutions. Lastly, optimization of resources use in flood management is also recommended frequently. In particular, optimization is suggested through better matching available finance to the planned action, and matching flood management problems to suitable expertise.

#### Strategy S5: Improve data and decision support

Strategy S5 consists of three solution clusters to improve data and decision support, namely supporting anticipatory flood management; addressing knowledge gaps and evaluating flood management measures. Firstly, experts commonly recommended anticipatory management based on effective and reliable data and decision support services. Specific improvements include improving flood monitoring; improving flood modelling; and developing effective forecasting and early warning systems. Furthermore, the experts also suggest to better synchronize data and to effectively deliver forecasting data to relevant users and regions. The second solution cluster focuses on addressing knowledge gaps through collecting more data and implementing integrated flood impact assessment. Regarding flood impact assessment, experts frequently focus on the impacts of hydropower dams along the Mekong’s mainstream on downstream flood hazard. The last solution cluster consists of two main solutions, namely testing measures before implementation and comparing different measures for implementation.

#### Strategy S6: Innovate and shift flood management approaches

Strategy S6 focuses on changes in flood management approaches at both operational and strategic levels. At the operational level, this strategy entails developing new technical measures and adapting current policies to better support flood management. Regarding new technical measures, the experts often suggest restoring the natural floodplains and developing flexible flood protection dikes to effectively distribute the flood water across the delta. At the strategic level, shifting the thinking and management paradigm is also often recommended. In particular, the experts suggest shifting from the conventional flood prevention approach towards integrated flood management using more diverse combinations of protection dikes with flood-resilience land-uses and livelihoods.

### Tailoring strategies and solutions to flood management challenges

We further analysed configurative aspects of the identified flood management solutions and strategies in relation to the challenges. This section presents findings along the two focal questions of (1) what are the main targeting challenges of each flood management strategies?, and (2) what are the combinations of flood management strategies and associated challenges to address flood management challenges in the Mekong Delta?

**Fig. 4:**
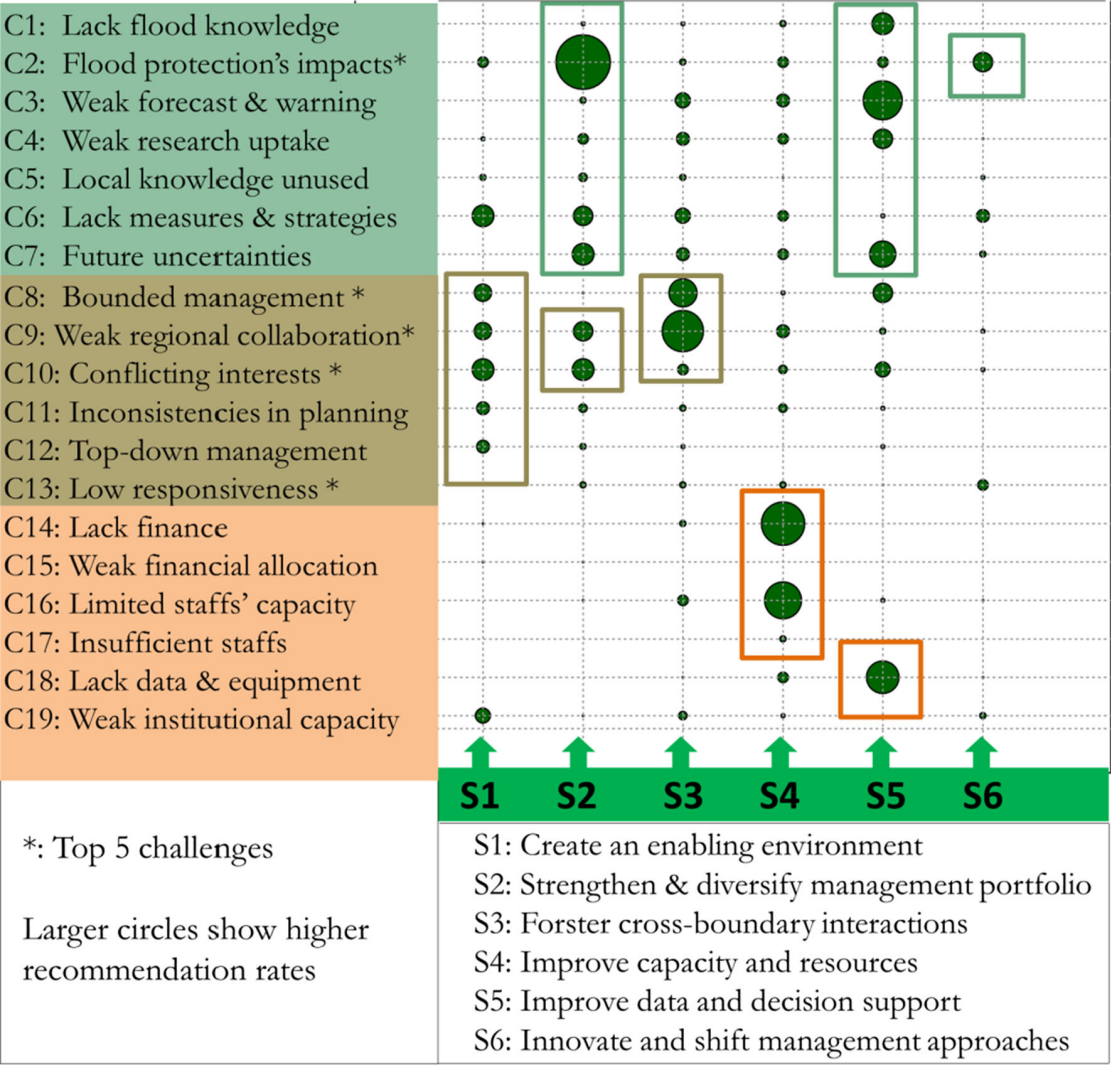
Tailoring strategies (S1–S6) to flood management challenges (C1–C19) based on expert survey. The circles show differentiated recommendation rates of the strategies to address each challenge. Full challenges’ description is available in Table 1

Figure 4 presents an overview of the linkages between challenges and flood management strategies based on calculated recommendation rates by our expert panel. Regarding targeting challenges of individual strategies, the differentiated recommendation rates (i.e. varying circle sizes) clearly indicate that the strategies and their associated solutions are tailored differently to the flood management challenges. In practical terms, this implies that while the strategies are highly suitable to address certain challenges (i.e. higher recommendation rates), they seem to be less applicable to others (i.e. lower recommendation rates). The recommendation patterns further show that individual strategies also target a specific group of flood management challenges. For example, strategy *S1*—*Create an enabling environment* mostly addresses challenges under the “Governance and institution” group. Similarly, strategy *S2*—*Enrich and strengthen the flood management portfolio* focuses strongly on “Technical” challenges, especially challenge C2 (i.e. unwanted impacts of existing flood protection measures).

We analysed the second aspect of the strategy-challenge configurations to understand how to combine multiple strategies to address the flood management challenges. The recommendation rates in Fig. 4 show that a majority of flood management challenges, including the top-three (i.e. C8, C9 and C10) require combining multiple response strategies and solutions. For example, the challenge of weak collaborations between different regions can be addressed through a combination of strategy *S3*—*Forster cross*-*boundary interactions*; *strategy S1*—*Creating an enabling environment* and strategy *S2*—*Strengthen and diversify the flood management portfolio*. For several challenges (e.g. C2, C9, C10), the recommendation rates per strategies also help to distinguish the primary and complementary strategies. Challenge C2 concerns unwanted impacts of the current flood protection dikes in the Mekong Delta. The recommendation pattern (Fig. 4) suggests that this challenge is primarily addressed through strategy *S2*—*Strengthen and diversify the flood management portfolio,* whereas strategy *S6*—*Innovate and shift management approach* can further complement S2.

Multiple flood management strategies should also be combined to address different challenge groups (i.e. technical; governance and institutional; and resources and capacity groups). In particular, three strategies (i.e. S2, S5 and S6) are recommended for the technical challenges group. The most important challenge in this group (i.e. *C2*—*Existing flood protection measures create unwanted impacts*) are linked with *S2*—*Enrich and strengthen flood management portfolio* and *S6*—*Innovate and shift approaches*. Similarly, the group of governance and institution challenges mostly require solutions under strategy *S1*—*Create an enabling environment*, strategy *S2*—*Enrich and strengthen flood management portfolio* and strategy *S3*—*Foster cross*-*boundary interactions*. For example, challenge *C9*—*Limited coordination and collaboration in flood management across provinces and districts* are tailored with “Develop a coordinating board for flood management”, “Promote exchange and learning” and “Improve collaboration between stakeholders”. Lastly, many solutions under the strategies S4 and S5 are regarded as relevant to address the group of resources and capacity challenges. Typical solutions for this challenge group include “Build capacity for flood management staff”, “Improve data sharing” and “Diversify funding sources”.

## Discussion

In this study, we have systematically identified a relatively large set of solutions to address emerging, yet critical flood management challenges in the Mekong River Delta. While many solutions are simultaneously tested and implemented, their integration and tailoring to specific challenges are often overlooked in both scientific and flood management domains (MDP 2013). We therefore analysed these configurative aspects to understand how individual solutions can be integrated, and how they are best tailored to specific challenges. New insights about such solution–solution and solution–challenge configurations can contribute to address serious limitations of the currently fragmented and infrastructure-centric flood management approach (Pham 2011). Below we discuss our main findings in relation to the existing literature and provide several policy recommendations.

We identified 19 flood management challenges, with about two-thirds of these challenges considered important by the expert panel. This further confirms that flood risks constitute a major threat to water-related safety in the Mekong delta (MDP 2013; Hoang et al. 2016). While many previous studies (Hoa et al. 2008; Kubiszewski et al. 2013; Piman et al. 2013) highlighted technical challenges, this study found that many critical challenges arise from the current governance and institutional settings. The strong focus on technical challenges is a logical reflection of the current technology-centric flood management approach. This approach, however, has become insufficient under the changing climate and accelerating socioeconomic developments, as suggested by our survey results, as well as by precious studies, including Käkönen (2008), Pham (2011), and Marchand et al. (2014). The existing governance and institutional settings have constrained the adoption of both ‘hard’ and ‘soft’ flood-risk management measures to transform parts of the current flood-risk management approach in order to effectively deal with future risks . This technical management approach, which is the result of path dependency caused by many past (investment) decisions, has probably created strong preferences over flood management practices being implemented in the Mekong Delta. In addition, the existing governance and institutional settings reinforce vested interests of actors and incentivize them to reinforce the status quo (Bachrach and Baratz 1970). This makes transformational changes (Kates et al. 2012) even more challenging, especially when these changes in the flood-risk management system should be fast, large scale and deep at the same time (Termeer et al. 2017).

We further found a large set (114 in total) of response solutions to address flood management challenges in the Mekong River Delta. The solutions’ diversity in terms of their quantity and multiple objectives reflect a complex flood management landscape as frequently reported in current literature (e.g. Birkmann et al. 2012; MDP 2013). In addition, diverse solutions emphasize the need to properly integrate and link these solutions to the challenges experienced in flood management. We found that the right configurations of response solutions and strategies are extremely important to address flood management challenges. The notion is especially relevant for the Mekong Delta where flood management is highly fragmented and documentations of solutions are scattered across different studies ("Current flood management approach and key challenges" section). While this study emphasizes integrated flood-risk management, its developed solution–solution and solution–challenge configurations further advance the currently underdeveloped configurative aspect of such management approach. In our focused Mekong Delta, we found that the current approach strongly relies on technical and infrastructural measures, and it has become insufficient under future higher flood risks and increasingly diversified, often contesting flood management objectives. The identified solutions and their configurations from this study demonstrate an alternative flood management approach, where technical and infrastructural measures are combined with, and thus supported by institutional and governance resolutions. This approach offers new possibilities to improve flood-risk management as well as to identifying interesting directions for further research.

Our findings for the Mekong River Delta about flood-risk management strategies and solutions can be applicable for other river basins in several aspects. First, we found that proper configurations of individual solutions are important for flood-risk management, especially when there are multiple, interconnected challenges ("Current flood management approach and key challenges" section, Supplementary Material S2-c). This finding is in line with those from several other cases, including the Bangladesh Delta (Brammer 2010) and the Duch Delta (van Staveren et al. 2014). For example, Brammer (2010) found that the full flood protection approach based solely on river and coastal embankments was infeasible and raised strong criticisms for the Bangladesh Delta. Secondly, we identified many flood management challenges emanating from the governance and institutional settings, which were also reported in other cases in Nepal (Dixit 2003) and Thailand (Lebel et al. 2011). Furthermore, this study reveals ‘soft’ measures as the oftentimes overlooked room for improvements in conventional flood management portfolios. While several studies advocate for transition from flood prevention towards ‘soft’ flood management approach (Wesselink et al. 2015; Liao et al. 2016), concrete solutions identified in this study can contribute to realize such transition.

Finally, we provide several recommendations for flood-risk management based on our findings. First, we recommend combining the strategies and solutions for implementation rather than deploying them individually. Whilst this seems self-evident, flood-risk measures are implemented in isolation and consequently face the challenge of becoming maladaptive, or create new challenges elsewhere (Lebel and Sinh 2009; Chapman et al. 2016). To effectuate transformational changes requires a more holistic approach that cannot be achieved by looking at individual challenges or implementing technical fixes in isolation. As most flood-risk challenges are co-occurring and intractably interlinked, they need to be simultaneously addressed to consider possible trade-offs. Second, given the challenges’ different manifestations across different spatial levels, adapting the strategies and solutions to the regional contexts is highly important for successful implementation. The identified challenges and solutions found in this study probably require further specification to operationalize and implement them. One possibility to do this is to organize stakeholder workshops to develop solution packages, targeting specific sets of challenges. Such approach can be useful to develop local flood management solutions that are relevant to the specific challenges and stakeholders’ needs.

## Conclusion

Effective flood-risk management is a top priority in the Vietnamese Mekong Delta. However, this process is increasingly challenged by climate change and accelerating socioeconomic developments. This is one of the first studies to systematically identify key challenges and to develop tailored intervention solutions and strategies, looking specifically at the rapidly changing flood management contexts under climate change and developments. We found that the challenges for flood management are diverse and multifaceted; however, many critical challenges predominantly arise from the current governance and institutional settings. We further identified a mismatch between the predominant governance and institutional challenges versus the conventional flood management approach, which strongly relies on technical and infrastructural measures. Minimizing flood risks under such circumstance requires adapting the current flood management approach to better account for the key challenges. In this study, we have identified six strategies to meet such requirement, namely (S1) Create a more enabling environment for flood management; (S2) Strengthen and diversify the flood management portfolio; (S3) Foster cross-boundary interactions; (S4) Improve capacity and resources; (S5) Improve data and decision support; and (S6) Innovate and shift flood management approaches. These strategies and their associated solutions contribute to the emerging repertoire of interventions in the literature to deal with some of the profound challenges in contemporary flood-risk management. Finally, we conclude that effective flood-risk management under rapid environmental change requires to explicitly account for the changing flood management landscape while developing and implementing intervention measures and strategies. In the Mekong Delta, re-configuring the conventional technology-centric flood management portfolio is highly important. Such re-configurations should focus on institutional changes and innovative measures, which offer ample opportunities to minimize flood risks under climate change and accelerating socioeconomic developments.

## Electronic supplementary material

Below is the link to the electronic supplementary material.

Supplementary material 1 (PDF 1,333 kb)

## Footnotes

1 The “Doi Moi” policy was introduced by the Vietnamese government as a major economic reform, aiming to boost economic growths and stability.
