# Supplementary material for: Managing flood risks in the Mekong Delta: How to address emerging challenges under climate change and socioeconomic developments
Source: Ambio. 2018 Feb 24;47(6):635–49. doi: 10.1007/s13280-017-1009-4 (PMC6131129; doi:10.1007/s13280-017-1009-4)
Supplement: Supplementary file 1 — Supplementary material 1 (PDF 1,333 kb) [file 13280_2017_1009_MOESM1_ESM.pdf]

Ambio

Electronic Supplementary Material

This supplementary material has not been peer reviewed.

Title: **Managing flood risks in the Mekong Delta: How to address emerging challenges under climate change and socioeconomic developments**

Authors: Long Phi Hoang, Robbert Biesbroek, Van Pham Dang Tri, Matti Kummu, Michelle T.H. van Vliet, Rik Leemans, Pavel Kabat, Fulco Ludwig

Supplementary Material S1
Systematic literature review of flood management and challenges

ID	Tittle	Full reference	Publication Type	Year	Scope	Theme 1: Flood/river modelling, monitoring, early warning	Theme 2: Flood vulnerability, damage and risk	Theme 3: Flood protection	Theme 4: Building flood resilience	Theme 5: Evaluating and CBA of flood management strategies/options	Theme 6: Flood's social aspects, incl. governance, perception and behavioural response	Theme 7: Flood related CC adaptation	Technical challenges in flood management	Institutional and governance challenges in flood management	Resources and capacity challenges in flood management
**1**	Climate Change Impact and Adaptation Study in the Mekong Delta	ADB, Climate Change Impact and Adaptation Study in the Mekong Delta. 2011, Asian Development Bank: Manila, Philippines.	2. Reports & working paper	2011	2. Vietnamese Mekong delta	1. Yes	1. Yes	0. No	0. No	0. No	0. No	1. Yes	**1.** Data and information is still scattered, monitoring networks are insufficient and data quality is poor leading to difficulties in climate change adaptation. **2.** Climate impact assessment based on global climate model output entails substantial uncertainties. **3.** It is difficult to transfer knowledge and insights from climate and meteorological modelling practices to other actors in order to support climate change adaptation and resources management.	**1.** Lack of ability to develop coordinated strategies and policies due to overlap of responsibilities among government agencies as well as lack of integration and coordination.	**1.** Budgets for natural resources development and management are limited and have not met the demand of the sector
**2**	Climate Risks in the Mekong Delta Ca Mau and Kien Giang Provinces of Viet Nam	ADB, Climate Risks in the Mekong Delta: Ca Mau and Kien Giang Provinces of Viet Nam. 2013, Asian Development Bank.	2. Reports & working paper	2013	4. Provinces	1. Yes	1. Yes	0. No	0. No	0. No	0. No	0. No	**1.** Uncertain climate change projections leading to difficulties in impact quantification, including impacts on floods	#	#
**3**	Combined fluvial and pluvial urban flood hazard analysis: concept development and application to Can Tho city, Mekong Delta, Vietnam	Apel, H. et al., Combined fluvial and pluvial urban flood hazard analysis: concept development and application to Can Tho city, Mekong Delta, Vietnam. Natural Hazards & Earth System Sciences 2016 16.4.	1. Peer-reviewed paper	2016	5. Sub-province	1. Yes	1. Yes	0. No	0. No	0. No	0. No	0. No	**1.** Understanding and modelling capacity for complex flood events (combined pluvial and fluvial floods) remain limited. **2.** Detailed verification of modelling results is contrained by limited observation data, e.g. data is either unavailable or is not long enough.	**1.** low coordination of water resources and flood management of riparian countries affects flood hazards for downstream regions.	#
**4**	Flood impact in the Mekong Delta, Vietnam	Balica, S., et al., Flood impact in the Mekong Delta, Vietnam. Journal of Maps, 2014. 10(2): p. 257-268.	1. Peer-reviewed paper	2013	2. Vietnamese Mekong delta	1. Yes	1. Yes	0. No	0. No	0. No	0. No	0. No	**1.** Lack of data and understandings about flood vulnerability under rapid economic growth. **2.** Lack of data, especially socio-economic data for flood vulnerability mapping. **3.** Hydraulic models are incapable of simulating some relevant flood parameters. **4.** Uncertainties in future socio-economic development leading to difficulties in estimating flood vulnerability indicator values.	#	#
**5**	Climate risks and adaptation strategies in the Lower Mekong River basin	Bastakoti, R.C., et al., Climate risks and adaptation strategies in the Lower Mekong River basin. Regional environmental change, 2014. 14(1): p. 207-219.	1. Peer-reviewed paper	2014	4. Provinces	0. No	0. No	1. Yes	1. Yes	0. No	1. Yes	1. Yes	**1.** Insufficient knowledge to implement necessary management measures.	**1.** Lack of effective coordination between agencies	**1.** Lack finance to adopt and sustain local management initiatives.
**6**	Vulnerability, Coping and Adaptation to Water Related Hazards in the Vietnamese Mekong Delta	Birkmann, J., et al., Vulnerability, coping and adaptation to water related hazards in the Vietnamese Mekong Delta, in The Mekong Delta System. 2012, Springer. p. 245-289.	4. Book chapter	2012	2. Vietnamese Mekong delta	0. No	0. No	0. No	0. No	0. No	1. Yes	1. Yes	**1.** Little is known about to what extent control structures for risk reduction and climate change adaptation might have side-impacts on the communities. 2. Lack of comprehensive study to quantify the relative importance of different drivers on flood water level.	**1.** Institutional responsibilities are in many cases ambiguous, particularly with respect to the synchronisation between disaster risk reduction and climate change adaptation tasks and funds. 2. MARD and MoNRE have similar mandates and areas of interests, causing competition over resources, competencies and power.	#
**7**	Adaptation and development trade-offs: fluvial sediment deposition and the sustainability of rice-cropping in An Giang Province, Mekong Delta	Chapman, A. D. et al., Adaptation and development trade-offs: fluvial sediment deposition and the sustainability of rice-cropping in An Giang Province, Mekong Delta." Climatic Change 2016, 137.3-4, 593-608.	1. Peer-reviewed paper	2016	4. Provinces	0. No	0. No	1. Yes	0. No	1. Yes	0. No	1. Yes	**1.** High flood protection dikes caused side impact for rice production through elimination of the natural sediment supply during floods, as well as increasing flood risk further downstream.	**1.** There exist conflicts between agricultural development and adaptation objectives. **2.** Current flood management approach using high dikes further exacerbate the rich-poor devide. **3.** Long term investment in flood protection dikes reinforce path-dependency in flood management system.	#
**8**	The 2011 flood event in the Mekong Delta: preparedness, response, damage and recovery of private households and small businesses	Chinh, D.T. et al., The 2011 flood event in the Mekong Delta: preparedness, response, damage and recovery of private households and small businesses. Disasters 2016, 40.4, 753-778.	1. Peer-reviewed paper	2016	4. Provinces	0. No	1. Yes	0. No	0. No	0. No	0. No	0. No	**1.** Flood risks in the Mekong Delta can be only partially estimated due to limited data availability. **2.** Sound flood risk management strategies are mostly lacking due to limited data for decision support. **3.** Flood emergency responses at local and household level are ineffective due to lack of early warning system.	**1.** Flood risk management and adaptation are not sufficiently integrated.	**1.** Heavy financial burden of flood losses is a dominant factor hampering the recovery of flood-affected households.
**9**	Living with Floods: An Evaluation of the Resettlement Program of the Mekong Delta of Vietnam	Danh, V.T. and S. Mushtaq, Living with floods: an evaluation of the resettlement program of the Mekong Delta of Vietnam, in Environmental change and agricultural sustainability in the Mekong Delta. 2011, Springer. p. 181-204.	2. Reports & working paper	2011	4. Provinces	0. No	0. No	0. No	1. Yes	1. Yes	0. No	0. No	**1.** Flood adaptation strategies (i.e. living with flood) causes considerable side-impacts due to insufficient planning and implementation capacity	#	#
**10**	Migration and Displacement Triggered by Floods in the Mekong Delta	Dun, O., Migration and displacement triggered by floods in the Mekong Delta. International Migration, 2011. 49(1): p. 200-223.	1. Peer-reviewed paper	2011	2. Vietnamese Mekong delta	0. No	0. No	0. No	0. No	0. No	1. Yes	0. No	**1.** Lack of empirical data about impacts of flood on migration;	#	#
**11**	Flood hazard in the Mekong Delta – a probabilistic, bivariate, and non-stationary analysis with a short-termed future perspective	Dung, N., et al., Flood hazard in the Mekong Delta-a probabilistic, bivariate, and non-stationary analysis with a short-termed future perspective. Natural Hazards and Earth System Sciences Discussions, 2013. 1: p. 275-322.	1. Peer-reviewed paper	2013	2. Vietnamese Mekong delta	1. Yes	0. No	0. No	0. No	0. No	0. No	0. No	**1.** Projected hydrological changes in the Mekong basin is uncertain. **2.** Lack of detailed flood hazard and risk assessment. **3.** Data on elevation and flood control infrastructure is still limited, causing difficulties in flood hazard assessment.	#	#
**12**	Local and global knowledge on flood management: How can local knowledge contribute to resilience in the Mekong delta, Vietnam	Ehlert, J., Local and Global Knowledge on Flood Management − How Can Local Knowledge Contribute to Resilience in the Mekong Delta, Vietnam? . 2007: Center for Development Research, Bonn University.	2. Reports & working paper	2007	2. Vietnamese Mekong delta	0. No	0. No	0. No	1. Yes	0. No	1. Yes	0. No	**1.** Local knowledge on living and benefiting from flood is largely neglected and underestimated in flood management. **2.** It is difficult to go beyond the consulting stakeholders but to actually activate and use local knowledge for flood management.	**1.** Conflicting interest, strategies and approaches to flood management, i.e. living with flood and flood protection between farmers and the government.	#

**13**	Analysis and attribution of trends in water levels in the Vietnamese Mekong Delta	Fujihara, Y. et al. Analysis and attribution of trends in water levels in the Vietnamese Mekong Delta. Hydrological Processes 2016 30.6: 835-845.	1. Peer-reviewed paper	2016	2. Vietnamese Mekong delta	1. Yes	0. No	0. No	0. No	0. No	0. No	0. No	**1.** The physical mechanisms of rising water level in the Mekong Delta are not well understood, causing challenge to design flood mitigation measures.	#	#
**14**	A hydrological model for interprovincial water resource planning and management: A case study in the Long Xuyen Quadrangle, Mekong Delta, Vietnam	Hanington, P. et al., A hydrological model for interprovincial water resource planning and management: A case study in the Long Xuyen Quadrangle, Mekong Delta, Vietnam. Journal of Hydrology 2017 547: 1-9.	1. Peer-reviewed paper	2017	3. Regions	1. Yes	0. No	0. No	0. No	0. No	0. No	0. No	**1.** Ultilizing modelling results for decision support is challenging due to limited reliability caused by missing and low quality observation data. **2.** Modelling results to support decision making at the provincial and district levels is very limited.	#	#
**15**	Climate Change Impacts on the Mekong Delta	Haruyama, S., Climate change impacts on the Mekong River	2. Reports & working paper	2009	2. Vietnamese Mekong delta	1. Yes	0. No	0. No	0. No	0. No	0. No	0. No	**1.** Knowledge about mechanisms of flood propagation and inundation is still lacking, which requires efforts on numerical modelling and scenarios assessment. **2.** Observation data is lacking for planning flood adaptation and mitigation.	**1**. Local flood protection increases flood risk further downstream, particularly: higher embankments and strengthening of dikes and roads have become obstructions to flood drainage, thus increasing flood peaks.	#
**16**	Environmental Issues and Recent Infrastructure Development in the Mekong Delta: review, analysis and recommendations with particular reference to large-scale water control projects and the development of coastal areas	Hashimoto, T., Environmental Issues and Recent Infrastructure Development in the Mekong Delta: review, analysis and recommendations with particular reference to large-scale water control projects and the development of coastal areas. 2001: Australian Mekong Resource Centre.	2. Reports & working paper	2001	2. Vietnamese Mekong delta	0. No	0. No	1. Yes	1. Yes	1. Yes	0. No	0. No	**1.** Flood control structure creates substantial side-effects, including increased flood risk for non-protected areas, ecological fragmentation and reduced sedimentation in the flood plain. **2.** Lack of baseline and post-intervention monitoring, leading to poor understanding of environmental impacts of flood protection measures.	#	#
**17**	Farmers' perceptions of climate variability and barriers to adaptation: lessons learned from an exploratory study in Vietnam	Hoa, L.D. et al., Farmers' perceptions of climate variability and barriers to adaptation: lessons learned from an exploratory study in Vietnam. Mitigation and adaptation strategies for global change, 2014. 19(5): p. 531-548.	1. Peer-reviewed paper	2014	2. Vietnamese Mekong delta	0. No	0. No	0. No	0. No	0. No	1. Yes	1. Yes	**1.** Limited attention and thus knowledge about climate risk perception of farmers leading to difficulties in promoting adaptation. **2.** Limited information availability and accessibility on climate impacts for adaptation planning and implementation.	#	**1.** Lack of financial and material resources is a barrier to adaptation to climate change impacts.
**18**	Farmers' assessments of private adaptive measures to climate change and influential factors: a study in the Mekong Delta, Vietnam	Hoa, L.D., et al., Farmers' assessments of private adaptive measures to climate change and influential factors: a study in the Mekong Delta, Vietnam. Natural hazards, 2014. 71(1): p. 385-401.	1. Peer-reviewed paper	2014	5. Sub-province	0. No	0. No	0. No	0. No	0. No	1. Yes	1. Yes	**1.** Limited understanding of how farmers appraise their private adaptive measures and influential factors. **2.** The fact that weather and the hydrological regime seemed to become more unpredictable leads to difficulties in considering adaptation measures. **3.** There are a number of barriers to the adaptive decisions of local farmers such as land tenure, maladaptation, technical knowledge of adaptive measures, access to market, habit, perception of the importance of climate change and adaptation, social relationship, access to credit, demographic factors, access to climate information and health care services. **4.** Limited technical knowledge about adaptation measures is a barrier to climate adaptation.	**1.** Inefficiency of rural information channels and associations can be possible causes of limited adaptation efficiency.	**1.** Some adaptation measures are too costly for farmers.
**19**	Infrastructure effects on floods in the Mekong River Delta in Vietnam	Hoa, L.T.V., et al., Infrastructure effects on floods in the Mekong River Delta in Vietnam. Hydrological Processes, 2008. 22(9): p. 1359-1372.	1. Peer-reviewed paper	2008	2. Vietnamese Mekong delta	1. Yes	0. No	1. Yes	0. No	1. Yes	0. No	0. No	**1.** Lack of user-friendly hydraulic modelling suites for flood studies. 2. Complicated flood regime and dynamics leading to difficulties in flood simulation. 3. Lack of data for flood modelling	#	#
**20**	Modelling to support land and water management: experiences from the Mekong River Delta, Vietnam	Hoanh, C.T., et al., Modelling to support land and water management: experiences from the Mekong River Delta, Vietnam. Water international, 2012. 37(4): p. 408-426.	1. Peer-reviewed paper	2012	2. Vietnamese Mekong delta	1. Yes	0. No	0. No	0. No	1. Yes	0. No	0. No	**1.** It is challenging for modellers to update the rapidly changing hydraulic network in the flood model. **2.** High level of complexity and connectedness creates difficulties in capturing and interpreting effects of human interventions on the hydrological regime. **3.** Data availability is a main limiting factor for advancing hydraulic modelling capability in the Mekong delta.	#	**1.** Financial constrain for collecting and updating data for hydraulic/flood modelling
**21**	Floodplain hydrology of the Mekong Delta, Vietnam	Hung, N.N., et al., Floodplain hydrology of the mekong delta, vietnam. Hydrological Processes, 2012. 26(5): p. 674-686.	1. Peer-reviewed paper	2012	5. Sub-province	1. Yes	0. No	0. No	0. No	0. No	0. No	0. No	**1.** Inundation dynamics, which is urgently required for planning, is ill-studied, mainly due to limited data. **2.** Flood protection via dykes system is controversial since local people's livelihood is affected;	#	#
**22**	Urbanization and climate change impacts on future urban flooding in Can Tho city, Vietnam	Huong, H. and A. Pathirana, Urbanization and climate change impacts on future urban flooding in Can Tho city, Vietnam. Hydrology and Earth System Sciences, 2013. 17(1): p. 379-394.	1. Peer-reviewed paper	2013	4. Provinces	1. Yes	1. Yes	0. No	0. No	0. No	0. No	0. No	**1.** Lack of observed data leads to difficulty in urban flood modelling. **2.** Extreme rainfall and tropical storm events becomes more unpredictable. **3.** Uncertain sea-level rise projection leading to difficulties in quantifying flood risk. **4.** Current estimation of urban flood risk is not very helpful for adaptation planning due to limited capacity to account for future changes in the system;	#	#
**23**	Climate change impacts on water resources in the Mekong delta and adaptation measures	IMHEN, Climate change impacts on water resources in the Mekong delta and adaptation measures. 2010, Institute for Meteorology, Hydrology and Environment.	2. Reports & working paper	2010	2. Vietnamese Mekong delta	1. Yes	1. Yes	1. Yes	0. No	0. No	0. No	1. Yes	**1.** Uncertainties in climate change projection and upstream development trends creates difficulties for projecting future changes in hydrology, including flood in the Mekong delta.	#	#
**24**	Climate change in the Mekong delta: Climate scenarios, sea level rise and other effects	IMHEN, Climate change in the Mekong delta: Climate scenario's, sea level rise and other effects 2013, Institute for meteorology, hydrology, environment and natural resources, Hanoi, Vietnam.	2. Reports & working paper	2013	2. Vietnamese Mekong delta	0. No	1. Yes	0. No	0. No	0. No	0. No	1. Yes	**1.** Observed climatic data is limited, data at some stations are disrupted and this leads to difficulties in hydrological modelling. **2.** Additional factors need to be taken into account to simulate changes in flood and water level. **3.** Lack of data and information on more vulnerable regions and groups	**1.** Lack of legislative documents to support climate change adaptation. 2. Lack of guidance and mechanisms for collaboration between government departments.	#
**25**	Climate change adaptation in the Mekong delta	JICA, Climate change adaptation in the Mekong delta. 2013, Japan International Cooperation Agency.	2. Reports & working paper	2013	2. Vietnamese Mekong delta	1. Yes	1. Yes	1. Yes	0. No	1. Yes	0. No	1. Yes	**1.** Human resources can not meet technical needs to cope with climate change impacts, including flood. **2.** Knowledge dissemination to local level is not effective. **3.** Lack of flood forecasting systems. **4.** Lack of feasible planning strategies to address climate change impacts. **5.** Planning to address climate change impacts are facing uncertainties in future changes in the delta system such as river flow from upstream. **6.** Uncertainties relating to climate change and Mekong's future discharge requires frequent update and modifications of plans	**1.** Management capacity to cope with climate change impact is lacking. 2. Overlapping in responsible staffs;	**1.** Investment fund from the government for planning and implementing hydraulic infrastructure is lacking and not timely.

**26**	Mekong Delta at the Crossroads: More Control or Adaptation?	Käkönen, M., Mekong Delta at the crossroads: more control or adaptation? AMBIO: A Journal of the Human Environment, 2008. 37(3): p. 205-212.	1. Peer-reviewed paper	2008	2. Vietnamese Mekong delta	0. No	0. No	1. Yes	1. Yes	1. Yes	1. Yes	0. No	**1.** The modified distribution of water has changed the distribution of risks: new canals and new control structures bring floods and saline intrusion to new areas. **2.** Construction of flood prevention dikes causes important negative impacts, including higher cost for rice cultivation due to forgone sedimentation, forgone fishery resource and exacerbation of environmental pollution.	**1.** Due to unequal distribution of costs and benefits of water engineering investments, the poorest farmers and landless people are often the disadvantaged agents. **2.** Conflicting provincial interests in flood management	#
**27**	Flood Mapping and Flood Dynamics of the Mekong Delta: ENVISAT-ASAR-WSM Based Time Series Analyses	Kuenzer, C., et al., Flood mapping and flood dynamics of the Mekong Delta: ENVISAT-ASAR-WSM based time series analyses. Remote Sensing, 2013. 5(2): p. 687-715.	1. Peer-reviewed paper	2013	2. Vietnamese Mekong delta	1. Yes	0. No	1. Yes	0. No	0. No	0. No	0. No	**1.** Flood regime in the Mekong delta is very complex, which requires large amount of data to understand the dynamics and drivers. **2.** There are multiple flood drivers and this makes it difficult to understand flood dynamics and mechanisms.	#	#
**28**	Flood and Salinity Management in the Mekong Delta, Vietnam	Le, A.T., et al., Flood and salinity management in the Mekong Delta, Vietnam, in Challenges to sustainable development in the Mekong Delta: Regional and national policy issues and research needs, T.T. Be, B.T. Sinh, and F. Miller, Editors. 2007, The Sustainable Mekong Research Network (Sumernet): Bangkok, Thailand. p. 15-68.	4. Book chapter	2005	2. Vietnamese Mekong delta	1. Yes	1. Yes	0. No	0. No	0. No	0. No	1. Yes	**1.** Collecting and processing of routine hydrological and meteorological data is an expensive and difficult task. **2.** Flood protection dykes seem to have adverse impacts such as prolonging flood duration in areas further downstream, increasing pets and diseases, etc. **3.** There is an urgent need for research on transboundary water cooperation and on environmental problems, rather than only technical problems since knowledge in this aspect is limited. **4.** Impacts of upstream dams development is a growing concern and further research is required	**1.** It is difficult to reach consensus and collaboration between different agencies for flood and salinity management	#
**29**	Climate Change in the Mekong River Delta and Key Concerns on Future Climate Threats	Le, A.T.C., Suppakorn, Climate change in the Mekong River Delta and key concerns on future climate threats, in Environmental change and agricultural sustainability in the Mekong Delta, M. Stewart and P. Coclanis, Editors. 2011, Springer. p. 207-217.	4. Book chapter	2011	2. Vietnamese Mekong delta	1. Yes	1. Yes	0. No	0. No	0. No	0. No	1. Yes	**1.** Data and information on flood risk and vulnerability for specific sector is limited. **2.** Knowledge on the combined impacts of multiple drivers on hydrological change is still limited, especially socio-economic factors	#	#
**30**	The combined impact on the flooding in Vietnam's Mekong River delta of local man-made structures, sea level rise, and dams upstream in the river catchment	Le, T.V.H., et al., The combined impact on the flooding in Vietnam's Mekong River delta of local man-made structures, sea level rise, and dams upstream in the river catchment. Estuarine, Coastal and Shelf Science, 2007. 71(1): p. 110-116.	1. Peer-reviewed paper	2007	2. Vietnamese Mekong delta	1. Yes	0. No	0. No	0. No	1. Yes	0. No	0. No	**1.** Data and information on upstream hydropower dams development are limited and uncertain, causing difficulties for quantifying their impacts on hydrological regime in the delta.	#	#
**31**	Urban design principles for flood resilience: Learning from theecological wisdom of living with floods in theVietnamese Mekong Delta	Liao, K. et al., Urban design principles for flood resilience: Learning from the ecological wisdom of living with floods in the Vietnamese Mekong Delta. Landscape and Urban Planning 2016, 155: 69-78.	1. Peer-reviewed paper	2016	2. Vietnamese Mekong delta	0. No	0. No	0. No	1. Yes	0. No	0. No	0. No	**1.** Excessive reliance on flood control infrastructure increases vulnerability of urbans to flood risks.	**1.** Local actors tend to retain an negative perception about flood while they overlook flood benefits. This results in strong preferences for flood prevention rather than flood adaptation. **2.** The perception that the government is solely responsible for hazard mitigation is prevalent, prevents wider implementation of adaptation measures.	
**32**	Mekong Delta: Living with Water, But for How Long?	Marchand, M., D.Q. Pham, and T. Le, Mekong Delta: Living with water, but for how long? Built Environment, 2014. 40(2): p. 230-243.	1. Peer-reviewed paper	2014	3. Regions	0. No	0. No	1. Yes	1. Yes	0. No	0. No	1. Yes	**1.** Local knowledge for flood management is underused by governmental specialists.	**1.** Planning and implementation of flood management interventions are very much top-down, leading to limited choices for farmers on the field.	**1.** Huge investment is needed for implementing recommended adaptation measures to cope with salinity intrusion and flooding.
**33**	Mekong delta plan: Long-term vision and strategies for a safe, prosperous and sustainable delta	MDP, Mekong delta plan: Long-term vision and strategies for a safe, prosperous and sustainable delta. 2013, Ministry of Natural Resources and Environment (Vietnam), Ministry of Agriculture and Rural Development (Vietnam), Ministry of Infrastructure and Environment (the Netherlands).	3. Planning documents	2013	2. Vietnamese Mekong delta	0. No	1. Yes	1. Yes	1. Yes	0. No	0. No	1. Yes	**1.** Impacts of climate change and upstream development activities on Mekong flows lack substantial quantification. **2.** Socio-economic development in the Mekong delta including population growth and urbanisation, GDP growth and development of the agricultural, industrial and services are highly uncertain.	**1.** Lack of human resource with adequate knowledge and experience leads to difficulties in water and flood management, especially at provincial and local level. Integrated planning for land and water management is hampered by limited information exchange and lack of common space in sectoral interests	#
**34**	Water governance assessment: The case of the Mekong delta	MDRI, Water governance assessment: The case of the Mekong delta. 2011, Mekong delta development research institute and climate change research institute: Cantho City, Vietnam.	2. Reports & working paper	2011	2. Vietnamese Mekong delta	0. No	0. No	0. No	0. No	0. No	1. Yes	0. No	#	**1.** Overlaps in tasks and responsibility across and within ministries for water management. **2.** Weak coordination and cooperation among sectors and provinces. **3.** Cooperation amongst sectors, between central and local government, and amongst provinces in comprehensive and multi-purpose exploitation and use of water resources remains loose and inefficient. **4.** Existing hydraulic infrastructure are not optimally used due to poor management. **5.** Lack of human resource for flood and disaster management.	#
**35**	Environmental governance: A Mekong delta case study with downstream perspectives	Minh, L.Q., Environmental Governance: A Mekong Delta case study with downstream perspectives. World Resources Institute, 2001.	2. Reports & working paper	2001	2. Vietnamese Mekong delta	0. No	0. No	0. No	0. No	0. No	1. Yes	0. No	**1.** Due to the complexity of the delta system, and uncertainties in upstream development impacts on the delta, reliable information for policy makers is not available.	**1.** Balancing multiple interests and policies in water governance is not always feasible in the delta. 2. The role of provincial authorities in water resource governance is undermined. **3.** Inconsistencies and overlap between different laws that regulate water and related resources, such as water laws, forest laws, environmental protection laws, land laws, etc., can hinder achievements of the overall goals	#
**36**	Flood Protection Criteria for the Mekong Delta, Vietnam	MRC, Flood Protection Criteria for the Mekong Delta, Vietnam. 2009, Mekong River Commission: Vientiane, Laos PDR.	2. Reports & working paper	2009	2. Vietnamese Mekong delta	1. Yes	1. Yes	1. Yes	0. No	1. Yes	0. No	0. No	**1.** Data for flood damage estimate is incomplete, thus calculated values often underestimate the damage in reality. **2.** It is difficult to test engineering measures for flood risk management since technical details for the measures are not available.	#	#

**37**	Structural Measures & Flood Proofing in the Lower Mekong Basin: Strategic Directions for Integrated Flood Risk Management in Focal Areas	MRC, Strategic directions for integrated flood risk management in focal areas, in The flood management and mitigation program. 2009, Mekong River Commission: Vientiane, Laos PDR.	2. Reports & working paper	2009	2. Vietnamese Mekong delta	1. Yes	0. No	1. Yes	0. No	1. Yes	0. No	0. No	**1.** Side-impact of infrastructural flood management options (mainly dikes) is substantial, including impacts on ecosystem services and biodiversity, increased flood risk for adjacent areas and foregone flood benefits.	#	#
**38**	Flooding in Mekong River Delta, Viet Nam	Nguyen, H.N., K.T. Vu, and X.N. Nguyen, Flooding in Mekong River Delta, Viet Nam. Human development report, 2007: p. 23.	2. Reports & working paper	2007	2. Vietnamese Mekong delta	0. No	1. Yes	0. No	0. No	0. No	1. Yes	0. No	**1.** Lack of knowledge and education in coping with climate change impacts increases level of vulnerability.	**1.** Lack of collaborations between institutions engaged in flood management. **2.** Lack of regional coordination in flood management.	#
**39**	Measuring Household Resilience to Floods: a Case Study in the Vietnamese Mekong River Delta	Nguyen, K.V., H. James, and H. James, Measuring household resilience to floods: A case study in the Vietnamese Mekong river delta. Ecology and Society, 2013. 18(3): p. 13.	2. Reports & working paper	2013	5. Sub-province	0. No	0. No	0. No	1. Yes	0. No	1. Yes	1. Yes	**1.** Knowledge and understanding about flood resilience of households and individuals is very limited	#	#
**40**	Mekong delta flood in the past and present	Nguyen, Q.M., Mekong Delta Floods in the Past and Present, in Online. URL: http://www.mekonginfo.org/mrc_en/doclib.nsf/0/2178B55DB040C07687256B86005EE48.html. 2000, Colleague of Science, Vietnam National University.	2. Reports & working paper	2000	2. Vietnamese Mekong delta	1. Yes	1. Yes	0. No	0. No	0. No	0. No	0. No	**1.** Observation data suggests that extreme flood occurs more often, even in consecutive years of 1994, 1995 and 1996. This increased extreme flood frequency could not be explained by conventional knowledge about flood in the Mekong delta; this causes confusions for both scientists and policy makers. **2.** Infrastructure development in the delta, particularly road network and salt water control gates eventually become obstacles for flood water transfer to the sea. **3.** The exact cause of changing flood regime is still not adequately studied, causing difficulty for managing flood in the delta.	#	#
**41**	Situation Analysis: Plain of Reeds, Viet Nam.	Nguyen, X.V. and A.R. Wyatt, Situation analysis: plain of reeds, Viet Nam. 2006: Mekong Wetlands Biodiversity Conservation and Sustainable Use Programme.	2. Reports & working paper	2006	3. Regions	0. No	1. Yes	0. No	0. No	0. No	1. Yes	0. No	#	**1.** Weak institutional capacity and limited farmers' participation in decision making processes in climate change adaptation.	**1.** Limited access of farmers to financial sources for adaptation to climate change impacts, including floods
**42**	Planning and implementation of the dyke systems in the Mekong Delta, Vietnam	Pham, C.H., Planning and implementation of the Dyke systems in the Mekong delta, Vietnam. PhD thesis. Faculty of Mathematics and Natural Sciences, Rheinischen Friedrich-Wilhelms, University of Bonn, Bonn. 2011.	2. Reports & working paper	2011	4. Provinces	0. No	0. No	1. Yes	0. No	1. Yes	1. Yes	0. No	**1.** It is difficult to develop effective flood control measures that do not creates substantial negative side-impacts. **2.** Lack of knowledge on social aspects of flood management. **3.** Lack of capacity and technical expertise for flood management. **4.** The Mekong delta is a complex system and develops very quickly, making it difficult to address any problem at the delta level. **5.** Lack of human resources for flood management.	**1.** Stakeholder participation and coordination in implementing flood protection dykes is very limited. **2.** Conflicting interest in water resources/flood management between countries and regions in the delta. **3.** lack of transparency and democracy in dykes planning and implementation.	**1.** High investment costs for flood protection dykes
**43**	Flood management and development planning: The allocation of risk in the Mekong delta, Vietnam	Reis, N., Flood management and development planning: The allocation of risk in the Mekong delta, Vietnam, in Bonn International Graduate School for Development Research, Universität Bonn. 2007.	2. Reports & working paper	2007	2. Vietnamese Mekong delta	0. No	0. No	1. Yes	0. No	1. Yes	1. Yes	0. No	**1.** Lack of information for flood management. **2.** Lack of planning capacity and human resource. **3.** Risk perception not adequately addressed in flood management	**1.** Lack of coordination in flood management between and within government agencies. **2.** Inconsistencies between plans at different levels. **3.** Lack of stakeholders participation and transparency in decision making processes for flood management.	#
**44**	The Water-Development Nexus: Importance of Knowledge, Information and Cooperation in the Mekong Delta	Renaud, F.G. and C. Kuenzer, The water-development nexus: importance of knowledge, information and cooperation in the Mekong Delta, in The Mekong Delta System: Interdisciplinary Analyses of a River Delta, F.G. Renaud and C. Künzer, Editors. 2012, Springer. p. 445-458.	4. Book chapter	2012	2. Vietnamese Mekong delta	0. No	0. No	0. No	0. No	0. No	1. Yes	0. No	**1.** One critical challenge is to to produce, present and communicate scientific knowledge in a manner that it reaches stakeholders at all levels so that crucial information for the basin or the delta can be incorporated into decision making, these processes are still limited in the delta.	**1.** Lack of knowledge-sharing within and between institutions leading to limited knowledge transfer to policy implementation agencies. **2.** It is challenging to clarify and enforcing responsibilities and collaboration at various governance levels for integrated water management. **3.** Horizontal cooperation among ministries or other organisations remainsweak.	#
**45**	The dimensions of flooding pattern in the Mekong delta - A struggle in a changing climate	Roddar, L.D.l., Isabella The dimensions of flooding in the Mekong delta: A struggle in a changing climate. 2010, Department of peace and development, Linne University.	2. Reports & working paper	2010	2. Vietnamese Mekong delta	0. No	1. Yes	0. No	0. No	1. Yes	1. Yes	0. No	**1.** Government's law and policies alone are not sufficient to properly address flooding problem in the delta; **2.** Socio-economic data to understand climate induced migration is limited, especially with regard to more vulnerable, marginalized households. **3.** Flood management is becoming more challenging due to extra uncertainties relating to future climate change	**1.** Lack of coorperation between regions and involving organizations for flood management	**1.** Funding for flood management is lacking
**46**	Master plan for water resources management for the Vietnamese Mekong delta under climate change and sea-level rise contexts	SIWRP, Master plan for water resources management for the Vietnamese Mekong delta under climate change and sea-level rise contexts. 2013, Southern Institute for Water Resources Planning, Hochiminh City, Vietnam.	3. Planning documents	2013	2. Vietnamese Mekong delta	0. No	0. No	1. Yes	0. No	1. Yes	0. No	1. Yes	**1.** Lack of data on water distribution among river branches for reliable calculations and hydraulic simulations. **2.** Uncertainties in upstream development scenarios, especially hydropower dams construction. **3.** Observed sea-level rise data is too short for statistical analysis. **4.** Lack of bio-physical data is one major challenge for water/flood management in the Mekong delta. **5.** Incoming discharge from upstream is becoming increasingly uncertain.	#	**1.** Socio-economic development level is lagging behind other regions, creating difficulties for financing climate change adaptation.
**47**	Sea-Level Rise and Land Subsidence: Impacts on Flood Projections for the Mekong Delta's Largest City	Takagi, H. et al., Sea-level rise and land subsidence: impacts on flood projections for the Mekong Delta's largest city. Sustainability 2016, 8.9	1. Peer-reviewed paper	2016	4. Provinces	1. Yes	1. Yes	0. No	0. No	0. No	0. No	0. No	**1.** Flood risk assessment for the Mekong Delta requires taking into account land subsidence, however this remains a challenging task due to unavailable data.**2.** Improper planning and implementation of flood control infrastructures can increase flood risk for certain areas.	#	#
**48**	Ocean Tides and the Influence of Sea-Level Rise on Floods in Urban Areas of the Mekong Delta	Takagi, H., et al., Ocean tides and the influence of sea-level rise on floods in urban areas of the Mekong Delta. Journal of Flood Risk Management, 2015. 8(4): p. 292-300.	2. Reports & working paper	2015	2. Vietnamese Mekong delta	0. No	1. Yes	0. No	0. No	0. No	0. No	0. No	**1.** The impacts of expected increasing flood water level for urban areas in the delta is still not well understood.	#	#
**49**	Comparison of historical land-use change patterns and recommendations for flood plain developments in three delta regions in Southeast Asia	Thi, M.M., L.N. Gunawardhana, and S. Kazama, A comparison of historical land-use change patterns and recommendations for flood plain developments in three delta regions in Southeast Asia. Water international, 2012. 37(3): p. 218-235.	1. Peer-reviewed paper	2012	2. Vietnamese Mekong delta	0. No	1. Yes	0. No	0. No	0. No	0. No	0. No	**1.** Current flood prevention system for the Mekong delta seems not adequate, given huge damage caused by the extreme flood event in 2000.	#	**1.** Increasing flood protection level through infrastructure investment in the Mekong delta is not economically feasible

**50**	Understanding farmer production strategies in context of policies for adaptation to floods in Vietnam Case study at two communes, An Giang province, Vietnam	Tran, V.H., Understanding farmer production strategies in context of policies for adaptation to floods in Vietnam. 2011, Dept. of Urban and Rural Development, Swedish University of Agricultural Sciences.	2. Reports & working paper	2011	5. Sub-province	0. No	0. No	1. Yes	1. Yes	1. Yes	0. No	1. Yes	#	#	**1.** Lack of financial and other material resources impede farmers' adoption of adaptation measures.
**51**	Vulnerability to Flood in the Vietnamese Mekong Delta: Mapping and Uncertainty Assessment	Trung, N.H. and V.Q. Thanh, Vulnerability to flood in the Vietnamese Mekong Delta: mapping and uncertainty assessment. Journal of Environmental Science and Engineering. B, 2013. 2(4B): p. 229.	1. Peer-reviewed paper	2013	2. Vietnamese Mekong delta	1. Yes	1. Yes	0. No	0. No	0. No	0. No	0. No	**1.** Lack of detailed data for simulating flood at the accuracy level suitable for engineering purposes. **2.** Lack of socio-economic data for projecting future flood vulnerability. **3.** Major inaccuracies exists in flood modelling at the delta scale **4.** Hydraulic network and river bathymetry needs frequent update due to rapid development and human modification	#	#
**52**	Multi-level governance and adaptation to floods in the Mekong delta	Trung, N.H., et al., Multi-level governance and adaptation to floods in the Mekong Delta, in Governing the Mekong: engaging in the politics of knowledge, R. Daniel, L. Lebel, and K. Manorom, Editors. 2013, Strategic Information and Research Development Centre (SIRD): Petaling Jaya	4. Book chapter	2013	2. Vietnamese Mekong delta	0. No	0. No	0. No	0. No	1. Yes	1. Yes	0. No	**1.** Apart from substantial benefits, the full dike system in the Mekong delta also have negative impacts on agriculture production and the environment. **2.** Flood forecasting and early warning is very limited due to lack of technical capacity.	**1.** Adapting to flood is limited by weak collaboration between prc	#
**53**	Adaptation of land use and houses in the upper Mekong Delta's deep flooding area	Van, N.K., Adaptation of land use and houses in the upper Mekong Delta's deep flooding area. 2012, Department of Architecture, National University of Singapore.	2. Reports & working paper	2012	3. Regions	0. No	0. No	0. No	1. Yes	1. Yes	1. Yes	1. Yes	1. Lack of knowledge on future climate change, seal-level rise, tropical storm and upstream hydropower dams impact leads to difficulties in estimating flood timing and intensity. This difficulty poses challenges for designing specific adaptation strategies.	**1.** There is a lack of collaboration between regional authorities and organisations for better managing flood and other climate change impacts.	**1.** Lack of financial resources for implementing adaptation measures.
**54**	A study of the climate change impacts on fluvial flood propagation in the Vietnamese Mekong Delta	Van, P., et al., A study of the climate change impacts on fluvial flood propagation in the Vietnamese Mekong Delta. Hydrology and Earth System Sciences, 16 (12), 2012, 2012.	1. Peer-reviewed paper	2012	2. Vietnamese Mekong delta	1. Yes	0. No	0. No	0. No	0. No	0. No	0. No	**1**. Climate change projection scenarios are uncertain, leading to high degree of uncertainties in the flood modelling results. 2. Complex river network and lack of detailed bathymetry data leads to limitation in understanding flood dynamics;	#	#
**55**	Flow dynamics in the Long Xuyen Quadrangle under the impacts of full-dyke systems and sea level rise	Van, P.D.T., H.T. Nguyen, and T.T. Nguyen, Flow dynamics in the Long Xuyen Quadrangle under the impacts of full-dyke systems and sea level rise. Journal of Earth Science, 2012. 28: p. 205-214.	1. Peer-reviewed paper	2012	3. Regions	1. Yes	0. No	0. No	0. No	0. No	0. No	0. No	**1.** Due to lack of data, it is not possible to simulate overland flow. 2. Construction of full dike system makes water level to rise faster and reaches higher level during floods; this causes negative impacts on agriculture and aquaculture activities in the North-West area of An Giang.	#	#
**56**	Modelling climate change impacts on the flood pulse in the Lower Mekong floodplains	Västilä, K., et al., Modelling climate change impacts on the flood pulse in the Lower Mekong floodplains. Journal of Water and Climate Change, 2010. 1(1): p. 67-86.	1. Peer-reviewed paper	2010	2. Vietnamese Mekong delta	1. Yes	0. No	0. No	0. No	0. No	0. No	0. No	**1.** Hydraulic modelling, especially in the cases of 2D and 3D models is challenging due to lack of observed data	#	#
**57**	Sea-level rise affecting the Vietnamese Mekong delta: Water elevation in the flood season and implications for rice production	Wassmann, R., et al., Sea level rise affecting the Vietnamese Mekong Delta: water elevation in the flood season and implications for rice production. Climatic Change, 2004. 66(1 2): p. 89-107.	1. Peer-reviewed paper	2004	2. Vietnamese Mekong delta	1. Yes	1. Yes	0. No	0. No	0. No	0. No	0. No	**1.** Flood modelling to understand future changes in flood regime is challenging due to lack of data on water discharge and topography. **2.** Lack of site-specific data on flood pattern for impact assessment studies.	#	#
**58**	From the Field: Flood Disaster Mitigation in the Mekong Delta	Weichselgartner, J. From the field: flood disaster mitigation in the Mekong Delta. in Proceedings for the 7th European Sociological Association Conference,'Rethinking Inequalities', Torun, Poland. 2005.	2. Reports & working paper	2005	2. Vietnamese Mekong delta	0. No	0. No	1. Yes	1. Yes	0. No	0. No	0. No	**1.** It is difficult to develop flood management policies that fulfil multiple objectives, including flood protection, maximizing productive uses and conserving ecological values. **2.** Narrow focus on geophysical processes and exposure and a preference for technical fixes and structural measures without adequately integrating social, economic, political, and legislative contexts. **3.** Lack of simple and comprehensive methodologies or instruments for integrating flood risk management into development planning. **4.** Lack appropriate tools for analysing cost and benefits of flood mitigation measures	**1.** Weak legal, regulatory and institutional frameworks for flood risk management. **2.** Insufficient capacities for disaster mitigation. **3.** China – where several hydro-projects greatly impact the Mekong Basin's water balance – is not a member of the MRC. This causes severe problems for the downstream countries concerning data availability and reliability. **4.** Lack of integration and coordination across administrative boundary creates difficulties in flood management planning and implementation.	#
**59**	Water management in seasonal floodplains of the Mekong delta: A case study from four villages in Cambodia and Vietnam	Werthmann, C., Water Management in Seasonal Floodplains of the Mekong Delta: A case study from four villages in Cambodia and Vietnam. Consilience: The Journal of Sustainable Development, 2010(3): p. 139-158.	1. Peer-reviewed paper	2010	5. Sub-province	0. No	0. No	0. No	1. Yes	0. No	1. Yes	0. No	#	**1.** Lack of coordination in flood/water management between governmental departments. 2. Lack/weak institutional support from government for flood/water management.	#
**60**	Water Management in the Mekong Delta: Changes, Conflicts and Opportunities	White, I., Water management in the Mekong Delta: changes, conflicts and opportunities. 2002, Unesco, Paris.	2. Reports & working paper	2002	2. Vietnamese Mekong delta	0. No	0. No	0. No	0. No	1. Yes	0. No	0. No	**1.** The control of downstream flooding and of saline intrusion in the lower Delta could be potentially in conflict with the need to reduce flooding in the upper Delta.	#	#

Supplementary Material S2: Expert survey and analyses

Supplement S2-a: Flood management in the Vietnamese Mekong delta: Identify challenges and explore solutions

Introduction

We welcome and thank you very much for taking your time to participate in our online-survey!

The objective of this survey is to draw on knowledge and experience of experts to gain better understanding about the challenges for flood risk management and explore possible solutions to address these challenges in the Vietnamese Mekong River Delta (hereafter the Mekong delta).

Throughout the survey, you will be asked to provide your expert judgements and recommendations on various aspects of flood management challenges. The questions are in multiple choice and open-ended formats. We would appreciate it very much if you provide detailed and specific answers to the open-ended questions. This would help us to draw meaningful conclusions from analysing the survey results. The survey takes approximately 15 minutes.

Thank you in advance for your support in our research!

The research team

I. General perspective on flood risk management and challenges

Q1. The Mekong delta has a long history of managing flood risk. To what extent do you agree with the following statement: "*Flood management in the Mekong delta has become more challenging compared to 30 years ago*"?

1. Fully agree
2. Agree
3. Neutral
4. Disagree
5. Strongly disagree
6. No answer

Q2. Literature has suggested several processes that make flood management more challenging. Based on your experience, please indicate the process(es) that make flood management in the Mekong delta more challenging compared to 30 years ago?

1. Climate change
2. Sea-level rise
3. Land use changes including deforestation in upstream countries
4. Hydropower dams construction in upstream countries
5. Population growth and urbanisation in upstream countries
6. Population growth and urbanisation in the Mekong delta
7. Dikes construction in the Mekong delta
8. Other process, namely: ....

Q3. Have you participated in any project concerning flood management in the Mekong delta? If yes, please give one project tittle.

Open answer:

....

II. Identifying important flood management challenges

Literature has identified many flood management challenges. They can be divided into three clusters, namely (i) *Knowledge and technical challenges*, (ii) *Institutional and governance challenges* and (iii) *resource challenges*. This section aims to find out the most important flood management challenges in the Mekong delta.

II-A. Technical challenges

Q4. Based on your experience, please indicate the importance of the following technical challenges in flood management in the Mekong delta? Please select the level of importance for each challenge.

**G1 - Technical challenges**	Very important	Important	Neutral	Unimportant	Very unimportant	No answer
C1: Lack of knowledge and understandings about the flood mechanisms in the floodplain						
C2: Existing flood protection measures create unintended impacts						
C3: Flood forecasting and early warning systems are not effective and reliable						
C4: Research results are not taken up in flood management processes						
C5: Local, indigenous knowledge is underused in flood management						
C6: Suitable strategies and measures for flood management are not available						
C7: Uncertainties in future climate change, sea-level rise and socio-economic development create difficulties for developing flood management plans						

II-B. Institutional and governance challenges

Q5. Based on your experience, please indicate the importance of the following institutional and governance challenges in flood management in the Mekong delta? Please select the level of importance for each challenge.

**G2 - Institutional and governance challenges**	Very important	Important	Neutral	Unimportant	Very unimportant	No answer
C8: Some factors causing flood are outside management boundary, i.e. in other country, province or district						
C9: Limited coordination and collaboration in flood management across provinces and districts						
C10: Conflicting interests between different management departments and regions						
C11: Flood and water management plans at different levels are inconsistent, leading to difficulties in implementation						
C12: Top-down, centralised approach to flood management						
C13: Flood management system is not responsive to new issues and challenges						

II-C. Resource challenges

Q6. Based on your personal experience, please indicate the importance of the following resource challenges in flood management in the Mekong delta? Please select the level of importance for each challenge.

**G3 - Resource & Capacity challenges**	Very important	Important	Neutral	Unimportant	Very unimportant	No answer
C14: Flood management lacks financial resource						
C15: Finance for flood management does not reach relevant regions and actors						
C16: Flood management staffs lack important capacities						
C17: Insufficient number of staffs for flood management						
C18: Lack of data and equipment for flood risk management						
C19: Limited institutional capacities for flood management, e.g. missing legislative instruments						

Q7. Apart from the above mentioned challenges, do you experience any other important flood management challenge(s)?

Open answer:

....
....

Q8. In previous questions, you have ranked the following challenges as important or very important. Please select <u>03 challenges</u> that you think are most important and thus need to be addressed so as to allow for improved flood risk management in the Mekong Delta.

Challenge 1 □

Challenge 2 □

....

Challenge n □

III. Explore solutions to address flood management challenges

In this section, we ask for your recommendations on solutions to overcome the most important flood management challenges in the Mekong delta.

Q9. In the previous step, you identified [FILL CHALLENGE] as one important flood management challenge. Could you please recommend two specific solutions, preferably with concrete examples, to overcome this challenge?

Open answer:

Solution 1:
....

Solution 2:
....

Q10. In the previous step, you identified [FILL CHALLENGE] as one important flood management challenge. Could you please recommend two specific solutions, preferably with concrete examples, to overcome this challenge?

Open answer:

Solution 1:
....

Solution 2:
....

Q11. In the previous step, you identified [FILL CHALLENGE] as one important flood management challenge. Could you please recommend two specific solutions, preferably with concrete examples, to overcome this challenge?

Open answer:

Solution 1:
....

Solution 2:
....

IV. Explore flood prevention measures

Flood risk can be mitigated through a number of flood prevention measures. In this section, we ask for your opinions on feasible infrastructure measures for flood prevention in the Mekong delta.

Q12. Based on your experience, please indicate which flood prevention measures are more relevant for the Mekong delta?

**Flood prevention measures**	Very relevant	Relevant	Neutral	Irrelevant	Very irrelevant	No answer
**1.** Controlled flooding in the Plain of Reeds and Long Xuyen Quadrangle. Agricultural land in these areas could be flooded to protect urbans.						
**2.** Full flood control for major cities and towns through improving and building new dikes.						
**3.** Creating retention zones and widen floodplains to store excessive flood water						
**4.** Improve existing flood water transfer capacity through river dredging, optimizing sluices/gates operation, etc.						
**5.** Build emergency flood diversion channels from Plain of Reeds and Long Xuyen Quadrangle to West and East Seas.						

Q13. Apart from the above mentioned measures, do you recommend any other infrastructure measures for flood protection in the Mekong delta?

Open answer:

....
....

V. Closing session

To finalise this survey, we would like to ask questions about your professional background. We only use the answers for analytical purpose and will only publish aggregated data.

Q14. Which of the below item best describe your occupation? Please select one item from the list below.

1. Government officer
2. Non-governmental organisation
3. Business/company
4. Social scientist
5. Natural scientist
6. Engineer
7. Other: ....

Q15. At which level is your work most focused on? Please select one item from the list below.

1. International
2. National
3. Regional (e.g. the Mekong delta)
4. Provincial
5. Municipal
6. Other: ....

Q16. Which of the following aspects of flood is your work most focused on? Please select one item from the list below

1. Flood research
2. Water management and planning
3. Land use management and planning
4. Flood protection infrastructures
5. Building flood resilience, living with flood
6. Climate change adaptation relating to flood
7. Flood early warning and emergency response
8. Other: ....

Q17. Is flood the most important component of your daily work?

1. Yes
2. No

Q18. What is your age category?

1. < 25 years old
2. 26 – 35 years old
3. 36 – 45 years old
4. 46 – 55 years old
5. 56 – 65 years old
6. > 65 years old

Q19. If you have any further comments/remarks about this questionnaire, please fill in the below lines.

....

Q20. You have finished our survey. We thank you very much for filling in the questionnaire!

Please indicate if you wish to receive the result of this survey:

1. Yes, please send results to ....
2. No thank you.

--------------------END----------------------

Supplement S2-b: Equations for calculating the flood management challenges' important levels and relating statistics

Aggregated important score

$$\text{Si} = \frac{\sum_{e=1}^{71} \text{Se,i}}{Ne} \qquad \text{(eq.1)}$$

Where

Si: Aggregated important score of challenge i; with i = 1:19
Se,i: Important score given by expert e to challenge i; with e = 1:71
Ne: Total number of expert; Ne=71

Group-wise important score

$$\text{Si, g} = \frac{\sum_{e=1}^{Ne,g} \text{Se, i}, g}{Ne, g} \qquad \text{(eq. 2)}$$

Where

Si,g: Aggregated important score of challenge i for expert group g; with i = 1:19. Experts were grouped according to their occupations; spatial working levels and working focus in relation to flood management.
Se,i,g: Important score given by expert e of group g to challenge i
Ne,g: Total number of expert in group g

Correlation coefficient between the challenges' ranking scores

$$\text{rx, y} = \frac{\sum_{i=1}^{n}(Xi - X)(Yi - Y)}{\sqrt{\sum_{i=1}^{n}(Xi - X)^2}\sqrt{\sum_{i=1}^{n}(Yi - Y)^2}} \qquad (eq.3)$$

Where

rx,y: Person correlation coefficient between important scores of challenge x and y
Xi: Individual experts' scores for challenge x
Yi: Individual experts' scores for challenge y
X: Average experts' score for challenge x
Y: Average experts' score for challenge y
n: Total numbers of scores given to each challenge; n=71

Supplement S2-c: Correlation coefficients between the challenge's rankings

	C1	C2	C3	C4	C5	C6	C7	C8	C9	C10	C11	C12	C13	C14	C15	C16	C17	C18	C19
**C1**	1	0.02	0.21	0.12	0.12	-0.1	-0.09	0.09	-0	0.08	**0.34****	0.02	0.17	-0	0.03	0.2	0.15	**0.27***	0.04
**C2**		1	0.1	0.03	0.04	0.14	0	0.01	0.15	**0.26***	**0.28***	0.08	0.1	0.09	**0.29***	0.03	-0.01	0.14	0.15
**C3**			1	0.09	0.2	**0.26***	0.07	0.06	-0.1	-0.1	0.19	0	0.2	**0.3***	0.18	**0.28***	**0.31****	**0.29***	0.16
**C4**				1	0.17	0.03	0.02	-0	0.09	0.13	0.13	0.16	0.05	0	-0.1	-0.09	-0.11	0.12	0.06
**C5**					1	0.22	**0.42****	0.07	**0.29***	0.2	**0.25***	**0.35****	**0.29***	-0	0.18	**0.32****	0.2	**0.38****	0.17
**C6**						1	**0.46****	-0.1	0.19	0.12	0.16	0.17	0.01	0.2	0.17	0.12	0.04	**0.33****	**0.4****
**C7**							1	0.21	**0.25***	0.13	0.19	0.04	**0.26***	0.1	-0.1	**0.3***	0.11	**0.32****	**0.29***
**C8**								1	-0.1	-0.04	0.12	0.19	**0.33****	-0	-0	0.13	0.18	0.05	-0.07
**C9**									1	**0.54****	**0.35****	0.12	**0.24***	0	**0.26***	0.08	0.07	0.22	**0.26***
**C10**										1	**0.4****	0.12	0.18	0.01	**0.38****	0.08	0.2	0.3	0.19
**C11**											1	0.13	0.13	0.18	0.2	**0.32****	**0.34****	**0.44****	**0.24***
**C12**												1	0.12	-0	0.08	-0.05	0.14	0.05	-0.05
**C13**													1	0.01	0.14	**0.27***	0.13	0.21	0.23
**C14**														1	0.21	**0.28***	**0.47****	0.23	0.18
**C15**															1	**0.29***	0.2	0.23	**0.32****
**C16**																1	**0.63****	**0.42****	0.21
**C17**																	1	**0.35****	**0.24***
**C18**																		1	**0.46****
**C19**																			**1**

*: Significant correlation at 0.95 confident level
**: Significant correlation at 0.99 confident level

Supplementary Material S3: Flood management solutions and strategies

Supplementary material S3-a: Inventory of the solutions to address flood management challenges

ID	Solutions	Recommendation frequency	Thematic strategy
**1**	Promote exchange and learning	24	Forster cross-boundary interactions
**2**	Implement integrated flood impact assessment	22	Improve data and decision support
**3**	Improve collaboration between actors	21	Forster cross-boundary interactions
**4**	Build capacity for flood management staff	21	Improve capacity and resources
**5**	Develop new technical measures	19	Innovate and shift flood management approaches
**6**	Improve communication	19	Forster cross-boundary interactions
**7**	Improve data sharing	16	Forster cross-boundary interactions
**8**	Improve collaboration between regions	15	Forster cross-boundary interactions
**9**	Revise existing measures	14	Strengthen and diversify the flood management portfolio
**10**	Improve human resources capacity	11	Improve capacity and resources
**11**	Promote participatory approach	11	Create an enabling environment for flood management
**12**	Promote integrated management	10	Strengthen and diversify the flood management portfolio
**13**	Develop new legislation	9	Create an enabling environment for flood management
**14**	Develop coordinating board	9	Create an enabling environment for flood management
**15**	Improve monitoring and early warning	9	Improve data and decision support
**16**	Shift thinking and management paradigm	8	Innovate and shift flood management approaches
**17**	Improve data's accuracy	8	Improve data and decision support
**18**	Improve coordination between regions	8	Create an enabling environment for flood management
**19**	Match expertise with problem	7	Improve capacity and resources
**20**	Generate funding from international collaboration	7	Improve capacity and resources
**21**	Improve institutional capacity	6	Improve capacity and resources
**22**	Improve coordination within region	6	Create an enabling environment for flood management
**23**	Develop agreements between regions	6	Create an enabling environment for flood management
**24**	Promote multi-objective flood management	6	Strengthen and diversify the flood management portfolio
**25**	Localize flood management	6	Strengthen and diversify the flood management portfolio
**26**	Set priorities in management	6	Strengthen and diversify the flood management portfolio

**27**	Improve flood modelling	5	Improve data and decision support
**28**	Generate funding from state budget	5	Improve capacity and resources
**29**	Diversify funding sources	5	Improve capacity and resources
**30**	Centralize flood management	5	Strengthen and diversify the flood management portfolio
**31**	Develop flood monitoring system	5	Improve data and decision support
**32**	Account for local conditions and resources	5	Improve capacity and resources
**33**	Invest in equipment	4	Improve capacity and resources
**34**	Develop education programs	4	Improve capacity and resources
**35**	Address unwanted impacts of existing measures	4	Strengthen and diversify the flood management portfolio
**36**	Enforce existing legislation	4	Create an enabling environment for flood management
**37**	Explore flood benefits	4	Strengthen and diversify the flood management portfolio
**38**	Synchronize flood monitoring, forecast and decision making	4	Improve data and decision support
**39**	Support stakeholders negotiation	4	Create an enabling environment for flood management
**40**	Adapt current policies	4	Innovate and shift flood management approaches
**41**	Resolve conflicts	4	Create an enabling environment for flood management
**42**	Promote integrated planning	4	Strengthen and diversify the flood management portfolio
**43**	Collect more data	4	Improve data and decision support
**44**	Test measures	4	Improve data and decision support
**45**	Develop visions	4	Strengthen and diversify the flood management portfolio
**46**	Improve coordination between actors	3	Create an enabling environment for flood management
**47**	Develop flood control system	3	Strengthen and diversify the flood management portfolio
**48**	Improve investment	3	Improve capacity and resources
**49**	Integrate multiple measures	3	Strengthen and diversify the flood management portfolio
**50**	Increase project funding	3	Improve capacity and resources
**51**	Develop adaptive measures	3	Strengthen and diversify the flood management portfolio
**52**	Publish research results	3	Improve data and decision support
**53**	Apply local knowledge in management	3	Strengthen and diversify the flood management portfolio
**54**	Compensate for negative management impacts	3	Strengthen and diversify the flood management portfolio
**55**	Improve training and education	3	Improve capacity and resources
**56**	Promote applied researches	2	Improve data and decision support

**57**	Promote flood-resilient development	2	Strengthen and diversify the flood management portfolio
**58**	Develop international agreements	2	Create an enabling environment for flood management
**59**	Optimize existing control infrastructures	2	Strengthen and diversify the flood management portfolio
**60**	Promote multi-level management	2	Strengthen and diversify the flood management portfolio
**61**	Improve planning	2	Strengthen and diversify the flood management portfolio
**62**	Raise awareness	2	Improve capacity and resources
**63**	Develop no-regret measures	2	Strengthen and diversify the flood management portfolio
**64**	Build bottom-up organisations	2	Strengthen and diversify the flood management portfolio
**65**	Localize flood research	2	Strengthen and diversify the flood management portfolio
**66**	Improve employment conditions	2	Improve capacity and resources
**67**	Improve transparency in management	2	Create an enabling environment for flood management
**68**	Establish flood research organisation	2	Improve data and decision support
**69**	Establish multi-stakeholder platform	2	Create an enabling environment for flood management
**70**	Develop alternative livelihoods	2	Innovate and shift flood management approaches
**71**	Improve data accessibility	2	Improve data and decision support
**72**	Adopt scenario-based planning	1	Strengthen and diversify the flood management portfolio
**73**	Apply international standards	1	Create an enabling environment for flood management
**74**	Avoid ineffective investment	1	Improve capacity and resources
**75**	Assess impacts of flood management	1	Improve data and decision support
**76**	Avoid technological lock-in	1	Strengthen and diversify the flood management portfolio
**77**	Develop early warning systems	1	Improve data and decision support
**78**	Create common understanding	1	Create an enabling environment for flood management
**79**	Develop data and information system	1	Improve data and decision support
**80**	Develop decision support system	1	Improve data and decision support
**81**	Combine forecast with indigenous knowledge	1	Improve data and decision support
**82**	Clarify responsibilities	1	Create an enabling environment for flood management
**83**	Compare measures	1	Improve data and decision support
**84**	Combine grant and loan in funding	1	Improve capacity and resources
**85**	Promote intermediary organisations	1	Forster cross-boundary interactions
**86**	Monitor implementation process	1	Improve data and decision support
**87**	Provide information to local level	1	Forster cross-boundary interactions

**88**	Provide demos and examples for proposed measures	1	Improve data and decision support
**89**	Match flood management with other objectives	1	Strengthen and diversify the flood management portfolio
**90**	Integrate multiple data sources	1	Improve data and decision support
**91**	Mitigate climate change	1	Strengthen and diversify the flood management portfolio
**92**	Match measures with available resources	1	Improve capacity and resources
**93**	Reduce population pressure	1	Strengthen and diversify the flood management portfolio
**94**	Set protection level	1	Strengthen and diversify the flood management portfolio
**95**	Set priorities in funding	1	Improve capacity and resources
**96**	Upgrade and maintain existing infrastructures	1	Strengthen and diversify the flood management portfolio
**97**	Shift power balance between actors	1	Create an enabling environment for flood management
**98**	Separate flood management from other objectives	1	Innovate and shift flood management approaches
**99**	Remove institutional barriers	1	Create an enabling environment for flood management
**100**	Set priorities for most vulnerable regions	1	Strengthen and diversify the flood management portfolio
**101**	Separate technical and managerial training	1	Innovate and shift flood management approaches
**102**	Implement and enforce existing plans	1	Strengthen and diversify the flood management portfolio
**103**	Improve flood emergency responses	1	Strengthen and diversify the flood management portfolio
**104**	Improve financial resources	1	Improve capacity and resources
**105**	Identify knowledge demands	1	Improve data and decision support
**106**	Focus research on basin-wide issues	1	Improve data and decision support
**107**	Evaluate quality of research results	1	Improve data and decision support
**108**	Focus training and education on the junior staff	1	Improve capacity and resources
**109**	Focus research on local issues	1	Improve data and decision support
**110**	Improve research funding	1	Improve capacity and resources
**111**	Improve measures applicability	1	Strengthen and diversify the flood management portfolio
**112**	Improve knowledge uptake	1	Improve data and decision support
**113**	Improve independence of legal institutions	1	Create an enabling environment for flood management
**114**	Improve recruitment	1	Improve capacity and resources

Supplementary material S3-b: Flood management strategies and associated solutions

Strategy S1: Create an enabling environment for flood management		
**Member solutions**	**Recommendation frequency**	**Solution ID**
Promote participatory approach	11	11
Develop new legislation	9	13
Develop coordinating board	9	14
Improve coordination between regions	8	18
Improve coordination within region	6	22
Develop agreements between regions	6	23
Enforce existing legislation	4	36
Support stakeholders negotiation	4	39
Resolve conflicts	4	41
Improve coordination between actors	3	46
Develop international agreements	2	58
Improve transparency in management	2	67
Establish multi-stakeholder platform	2	69
Apply international standards	1	73
Create common understanding	1	78
Clarify responsibilities	1	82
Shift power balance between actors	1	97
Remove institutional barriers	1	99
Improve independence of legal institutions	1	113

Strategy S2: Strengthen and diversify the flood management portfolio		
**Member solutions**	**Recommendation frequency**	**Solution ID**
Revise existing measures	14	9
Promote integrated management	10	12
Promote multi-objective flood management	6	24
Localize flood management	6	25
Set priorities in management	6	26
Centralize flood management	5	30
Address unwanted impacts of existing measures	4	35
Explore flood benefits	4	37
Promote integrated planning	4	42
Develop visions	4	45
Develop flood control system	3	47
Integrate multiple measures	3	49
Develop adaptive measures	3	51
Apply local knowledge in management	3	53
Compensate for negative management impacts	3	54
Promote flood-resilient development	2	57
Optimize existing control infrastructures	2	59

Promote multi-level management	2	60
Improve planning	2	61
Develop no-regret measures	2	63
Build bottom-up organisations	2	64
Localize flood research	2	65
Adopt scenario-based planning	1	72
Avoid technological lock-in	1	76
Match flood management with other objectives	1	89
Mitigate climate change	1	91
Reduce population pressure	1	93
Set protection level	1	94
Upgrade and maintain existing infrastructures	1	96
Set priorities for most vulnerable regions	1	100
Implement and enforce existing plans	1	102
Improve flood emergency responses	1	103
Improve measures applicability	1	111

Strategy S3: Forster cross-boundary interactions

Member solutions	Recommendation frequency	Solution ID
Promote exchange and learning	24	1
Improve collaboration between actors	21	3
Improve communication	19	6
Improve data sharing	16	7
Improve collaboration between regions	15	8
Promote intermediary organisations	1	85
Provide information to local level	1	87

Strategy S4: Improve capacity and resources

Member solutions	Recommendation frequency	Solution ID
Build capacity for flood management staff	21	4
Improve human resources capacity	11	10
Match expertise with problem	7	19
Generate funding from international collaboration	7	20
Improve institutional capacity	6	21
Generate funding from state budget	5	28
Diversify funding sources	5	29
Account for local conditions and resources	5	32
Invest in equipment	4	33
Develop education programs	4	34
Improve investment	3	48
Increase project funding	3	50
Improve training and education	3	55
Raise awareness	2	62
Improve employment conditions	2	66

Avoid ineffective investment	1	74
Combine grant and loan in funding	1	84
Match measures with available resources	1	92
Set priorities in funding	1	95
Improve financial resources	1	104
Focus training and education on the junior staff	1	108
Improve research funding	1	110
Improve recruitment	1	114

Strategy S5: Improve data and decision support

**Member solutions**	**Recommendation frequency**	**Solution ID**
Implement integrated flood impact assessment	22	2
Improve monitoring and early warning	9	15
Improve data's accuracy	8	17
Improve flood modelling	5	27
Develop flood monitoring system	5	31
Synchronize flood monitoring, forecast and decision making	4	38
Collect more data	4	43
Test measures	4	44
Publish research results	3	52
Promote applied researches	2	56
Establish flood research organisation	2	68
Improve data accessibility	2	71
Assess impacts of flood management	1	75
Develop early warning systems	1	77
Develop data and information system	1	79
Develop decision support system	1	80
Combine forecast with indigenous knowledge	1	81
Compare measures	1	83
Monitor implementation process	1	86
Provide demos and examples for proposed measures	1	88
Integrate multiple data sources	1	90
Identify knowledge demands	1	105
Focus research on basin-wide issues	1	106
Evaluate quality of research results	1	107
Focus research on local issues	1	109
Improve knowledge uptake	1	112

Strategy 6: Innovate and shift approaches		
**Member solutions**	**Recommendation frequency**	**Solution ID**
Develop new technical measures	19	5
Shift thinking and management paradigm	8	16
Adapt current policies	4	40
Develop alternative livelihoods	2	70
Separate flood management from other objectives	1	98
Separate technical and managerial training	1	101
